# Regulation of miR-146a by RelA/NFkB and p53 in ST*Hdh^Q111^*/*Hdh^Q111^* Cells, a Cell Model of Huntington's Disease

**DOI:** 10.1371/journal.pone.0023837

**Published:** 2011-08-26

**Authors:** Jayeeta Ghose, Mithun Sinha, Eashita Das, Nihar R. Jana, Nitai P. Bhattacharyya

**Affiliations:** 1 Crystallography and Molecular Biology Division, Saha Institute of Nuclear Physics, Kolkata, West Bengal, India; 2 Structural Genomics Division, Saha Institute of Nuclear Physics, Kolkata, West Bengal, India; 3 Division of Cellular and Molecular Neuroscience, National Brain Research Centre, Manesar, Haryana, India; Hertie Institute for Clinical Brain Research and German Center for Neurodegenerative Diseases, Germany

## Abstract

Huntington's disease (HD) is caused by the expansion of N-terminal polymorphic poly Q stretch of the protein huntingtin (HTT). Deregulated microRNAs and loss of function of transcription factors recruited to mutant HTT aggregates could cause characteristic transcriptional deregulation associated with HD. We observed earlier that expressions of miR-125b, miR-146a and miR-150 are decreased in ST*Hdh^Q111^*/*Hdh^Q111^* cells, a model for HD in comparison to those of wild type ST*Hdh^Q7^/Hdh^Q7^* cells. In the present manuscript, we show by luciferase reporter assays and real time PCR that decreased miR-146a expression in ST*Hdh^Q111^*/*Hdh^Q111^* cells is due to decreased expression and activity of p65 subunit of NFkB (RelA/NFkB). By reporter luciferase assay, RT-PCR and western blot analysis, we also show that both miR-150 and miR-125b target p53. This partially explains the up regulation of p53 observed in HD. Elevated p53 interacts with RelA/NFkB, reduces its expression and activity and decreases the expression of miR-146a, while knocking down p53 increases RelA/NFkB and miR-146a expressions. We also demonstrate that expression of p53 is increased and levels of RelA/NFkB, miR-146a, miR-150 and miR-125b are decreased in striatum of R6/2 mice, a mouse model of HD and in cell models of HD. In a cell model, this effect could be reversed by exogenous expression of chaperone like proteins HYPK and Hsp70. We conclude that (i) miR-125b and miR-150 target p53, which in turn regulates RelA/NFkB and miR-146a expressions; (ii) reduced miR-125b and miR-150 expressions, increased p53 level and decreased RelA/NFkB and miR-146a expressions originate from mutant HTT (iii) p53 directly or indirectly regulates the expression of miR-146a. Our observation of interplay between transcription factors and miRNAs using HD cell model provides an important platform upon which further work is to be done to establish if such regulation plays any role in HD pathogenesis.

## Introduction

Huntington's disease (HD) is an autosomal dominant neurodegenerative disorder caused by the expansion of polymorphic CAG repeats in exon1 of Huntingtin (*HTT*) gene. Among various molecular and cellular dysfunctions originated from mutations to *HTT* gene, which eventually lead to neuronal loss from striatal regions in HD patients, transcriptional deregulation is considered to be one of the important events [1], [2]. Such deregulation of genes has been attributed, at least partially, to interactions and recruitments of several transcription factors to the mutant HTT aggregates [2], [3]. Transcription factors (TFs) like TBP, CBP, p53, Sp1, NFkB and others are recruited to aggregates formed by mutant HTT, the hallmark of HD [4]–[9]. Functional consequence of such recruitment remains largely unknown. Recruitment of TFs to the aggregates may result in loss of functions of the TFs. This can explain the altered expressions of many genes in HD [2], [3]. In the presence of mutated HTT exon1, repression of transcription from p53-responsive promoters is detected, indicating hypo function of p53 in HD [8]. However, the level of p53 is increased in various models of HD as well as in the affected tissue in HD patients possibly due to post transcriptional or post-translational modifications [4]. It has also been shown that p53 directly interacts with the promoter sequence of *HTT* gene that harbors multiple p53 response elements [10]. Increased expression of mutant HTT due to higher level of p53 in turn may increase the aggregates formed by mutant HTT. Direct evidence that p53 participates in the pathogenesis of HD is also available [11]. However, effects of recruitment and interaction of NFkB with mutant HTT in HD pathogenesis remains unclear. In a cell model of HD, it has been shown that NFkB activity is increased in the early stage when there are no visible aggregates of mutant HTT, while at a later stage when visible aggregates are formed, NFkB activity is reduced [12]. Similar decrease in NFkB activity after 72 hours of induction of mutant HTT was also observed in a cell model of HD, while in early stage of induction, NFkB activity was increased [13], [14]. This dual role of mutant HTT on NFkB activity could be due to initial protective action of NFkB, which is suppressed at a later stage by the recruitment of NFkB into the aggregates. Alteration of NFkB activity may result in altered expression of NFkB regulated genes.

Micro RNA (miRNA) belongs to a class of small non-coding single stranded RNA, approximately 21 nucleotides long, and generally regulates gene expression negatively. Mature miRNA interacts mostly with 3′ untranslated regions (3′UTRs) of the genes in human and down regulates the expression of the targets either by degrading the mRNAs or by inhibiting their translation. In some cases, increased expression of target genes by miRNAs have also been reported [15]. Recent experiments show that at least in few specific cases, mature miRNA can alter the expression of genes even by binding to the coding regions as well as to the 5′ UTRs of its targets [16], [17], [18]. It thus provides further complex regulation of genes by miRNAs. It has been proposed on the basis of theoretical analysis that as many as 30% of genes in the human genome may be the targets of miRNAs [19]. However, latter estimates predict that as large as 90% of human genes are targets of miRNAs [20], although experimentally validated targets are limited. MiRNA genes are regulated in similar way as that of coding genes [21], [22]. For example, p53 is known to increase as well as decrease the expression of several miRNAs [23]–[27]. Interestingly, p53 is one of the targets of miR-125b [28], which is itself negatively regulated by p53 [26]. RelA/NFkB regulates the expression of miR-146a [29]. The neuron-restrictive silencer factor (NRSF), also known as Repressor Element Silencing Transcription Factor (REST), another HTT interacting protein, regulates several miRNAs. Among them, miR-132, miR-124, miR-9 and miR-9* are down regulated in affected tissues of HD patients [30], [31]. To investigate whether miRNA expressions are altered in HD, we recently identified changes in expressions of several miRNAs in ST*Hdh^Q111^*/*Hdh^Q111^* cells, a cell model of HD. We also characterized that miR-146a which is down regulated in the cell model targets TBP [32], [33].

As RelA/NFkB regulates the expression of miR-146a [29], in the present manuscript, we first tested the hypothesis that down regulation of miR-146a could be due to decreased activity of NFkB in ST*Hdh^Q111^*/*Hdh^Q111^* cells. Further, we tested whether p53 is a target of miRNAs, which are down regulated in ST*Hdh^Q111^*/*Hdh^Q111^* cells [33]. We then focused on the regulation of miR-146a by both RelA/NFkB and p53. We observed that NFkB activity is compromised in ST*Hdh^Q111^*/*Hdh^Q111^* cells and exogenous expression of p65 sub-unit of NFkB i.e. RelA/NFkB increased the expression of mature miR-146a in ST*Hdh^Q111^*/*Hdh* ^Q111^ cells. In addition, we showed that increased level of p53 in ST*Hdh^Q111^*/*Hdh^Q111^* cells could be due to decreased level of miR-150 and miR-125b. Besides, we also showed that exogenous p53 decreased the expression of RelA/NFkB and also reduced NFkB activity. Besides, p53 directly or indirectly regulated the expression of miR-146a. Further, results obtained with mutant HTT aggregates led us to postulate that in the presence of the aggregates there is an initial decrease in miR-125b and miR-150 expression. These down regulated miRNAs lead to increased p53 level. Elevated p53 then in turn, may decrease RelA/NFkB expression, NFkB activity and miR-146a expression.

## Results

### Regulation of miR-146a by RelA/NFkB in STHdh^Q7^/Hdh^Q7^ and STHdh^Q111^/Hdh^Q111^ cells

**Figure 1:**
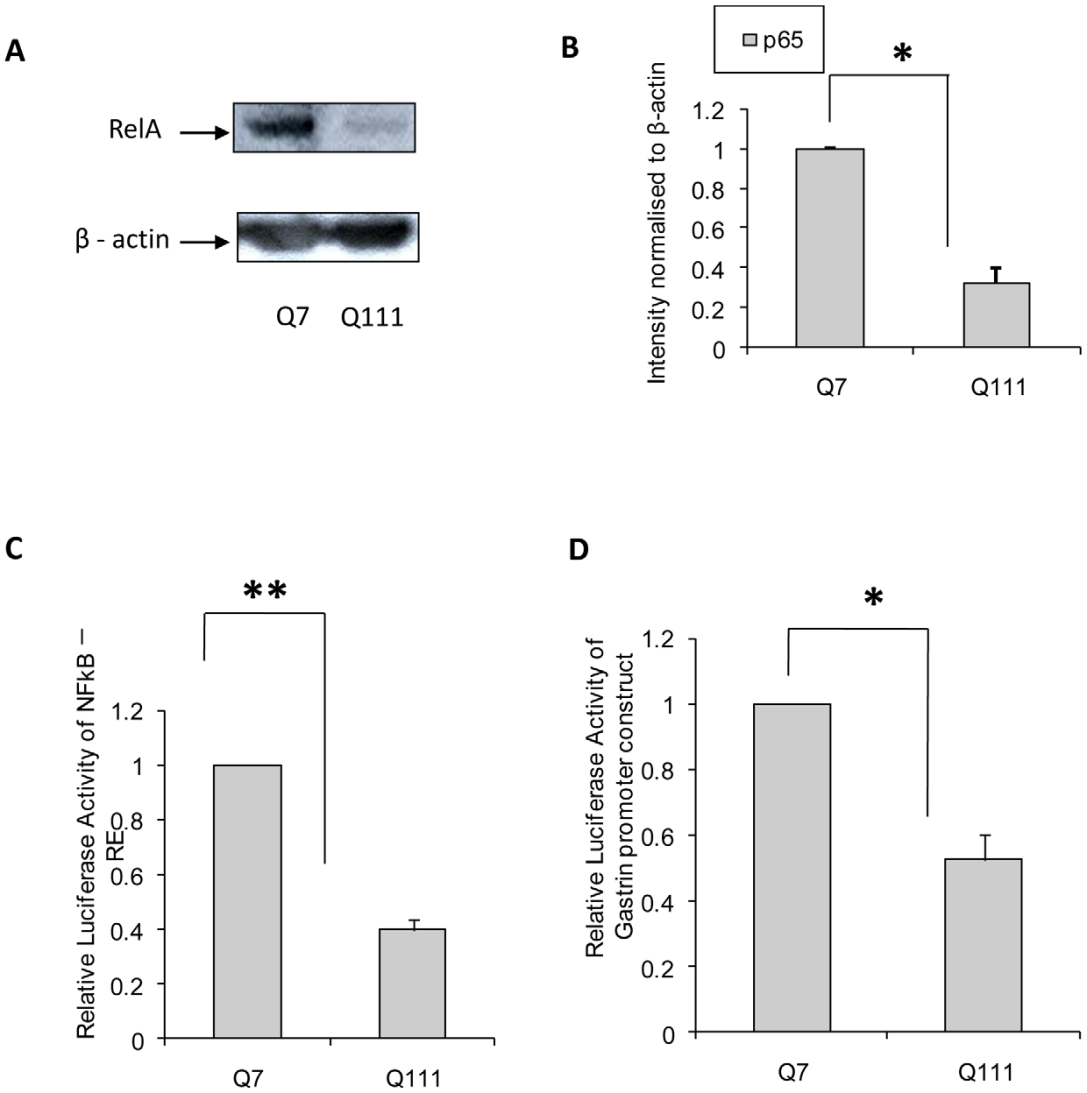
RelA/NFkB expression and activity in ST*Hdh^Q111^*/*Hdh^Q111^* and *S*T*Hdh^Q7^/Hdh^Q7^* cells. (**A**) Representative Western Blot showing decreased level of RelA/NFkB (denoted by p65 in the figure) in ST*Hdh^Q111^*/*Hdh^Q111^* cells compared to ST*Hdh^Q7^/Hdh^Q7^* cells; (**B**) Average integrated optical density (IOD) of p65 protein bands (RelA/NFkB) in *A*, normalized to β-actin level (n = 3, p = 0.018) in these cells; (**C**) Average luciferase activity using reporter luciferase with multiple NFkB response elements (denoted by NFkB-RE) in ST*Hdh^Q111^*/*Hdh^Q111^* cells compared to ST*Hdh^Q7^/Hdh^Q7^* cells. Normalization of protein level between ST*Hdh^Q7^/Hdh^Q7^* cells and ST*Hdh^Q111^/Hdh^Q111^* cells was done by taking the ratio of Relative Luciferase Units (RLU) of NFkB-RE and empty vector pGL3 in these cells. The normalized value obtained with ST*Hdh^Q7^/Hdh^Q7^* cells was taken as 1. Relative luciferase activity of NFkB-RE was found significantly lower (n = 4, p = 0.0082) in ST*Hdh^Q111^/Hdh^Q111^* cells compared to ST*Hdh^Q7^/Hdh^Q7^* cells; (**D**) Average reporter luciferase activity with Gastrin promoter (n = 2, p = 0.034) in ST*Hdh^Q7^/Hdh^Q7^* cells and ST*Hdh^Q111^*/*Hdh^Q111^* cells. Normalization of protein level between the cells was done by taking the ratio of Relative Luciferase Units (RLU) of Gastrin promoter construct and empty vector pGL3 in these cells. The normalized value obtained with ST*Hdh^Q7^/Hdh^Q7^* cells was taken as 1; Error bars represent standard deviation s of more than 2 experiments and each experiment was done in duplicate. “*” represents statistical significance; * p≤0.05; ** p<0.01.

We have shown earlier that expressions of several miRNAs are altered in ST*Hdh^Q111^/Hdh^Q111^* cells in comparison with ST*Hdh^Q7^/Hdh^Q7^* cells. Among the altered miRNAs, miR-146a is down regulated [33]. It is known that RelA/NFkB regulates the expression of miR-146a [29]. To investigate the possible role of RelA/NFkB in the observed down regulation of miR-146a in ST*Hdh^Q111^/Hdh^Q111^* cells, we first determined the steady state level (expression) of p65 sub-unit of NFkB i.e. RelA/NFkB in these cells. Western blot analysis revealed that the expression of RelA/NFkB (denoted by p65 in **Figure 1A**) was indeed decreased significantly (n = 3, p = 0.018) in ST*Hdh^Q111^/Hdh^Q111^* cells in comparison with that in ST*Hdh^Q7^/Hdh^Q7^* cells (**Figures 1A and 1B**). Using luciferase reporter assay with multiple NFkB responsive elements (denoted as NFkB-RE), we further observed that NFkB activity was also significantly (n = 4, p = 0.0082) compromised in ST*Hdh^Q111^/Hdh^Q111^* cells (**Figure 1C**). This was further confirmed by using gastrin promoter tagged reporter luciferase activity assay. It is known that RelA/NFkB regulates gastrin gene expression [34]. This result shown in **Figure 1D** confirms that NFkB activity is compromised in ST*Hdh^Q111^/Hdh^Q111^* cells (n = 2, p = 0.034).

**Figure 2:**
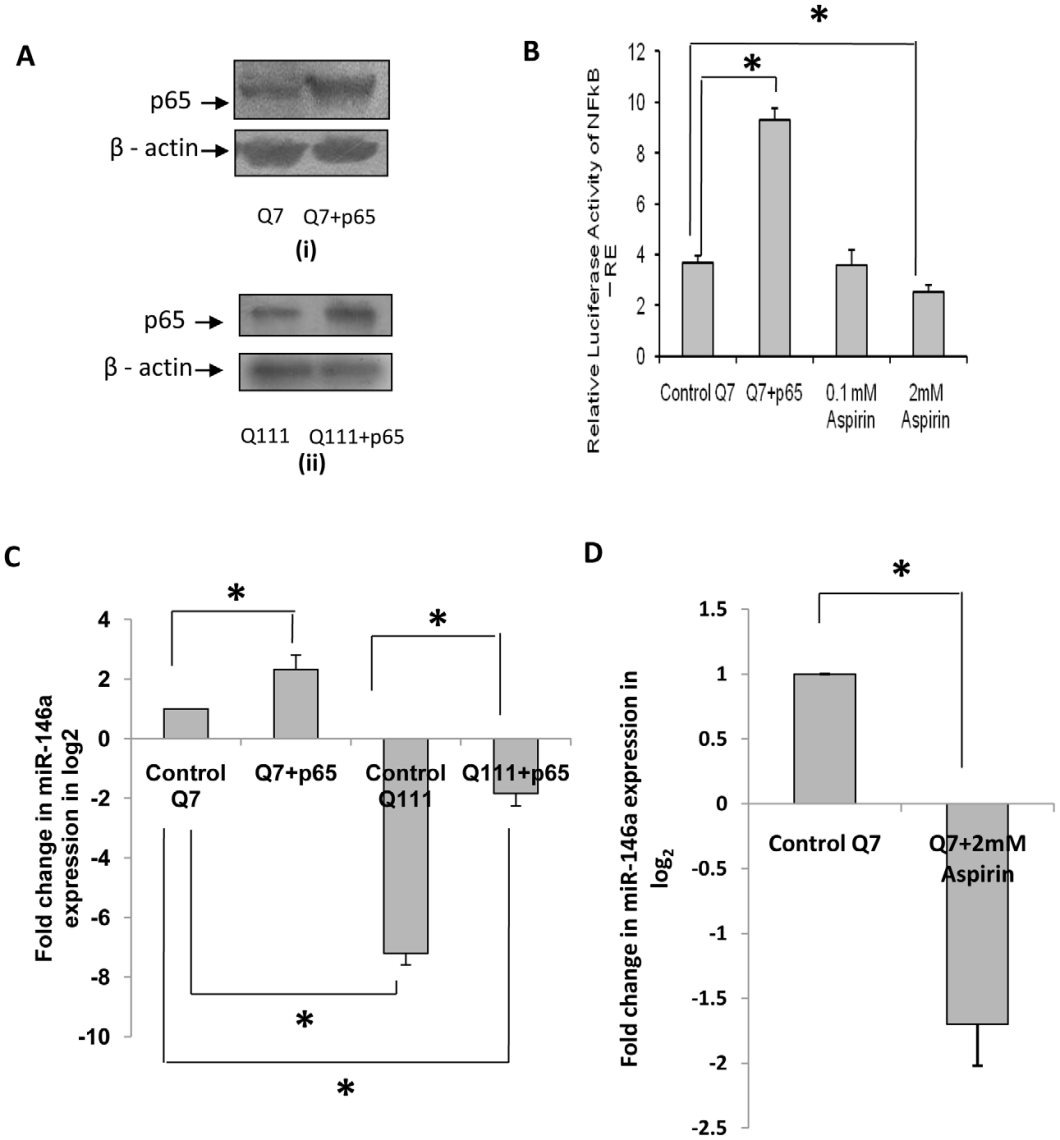
Regulation of miR-146a by RelA/NFkB in ST*Hdh^Q7^/Hdh^Q7^* and ST*Hdh^Q111^/Hdh^Q111^* cells. (**A**) Western Blot showing increased p65 protein (RelA/NFkB) level in (i) ST*Hdh^Q7^/Hdh^Q7^* cells expressing exogenous p65 subunit of NFkB (RelA/NFkB) compared to control ST*Hdh^Q7^/Hdh^Q7^* cells and in (ii) ST*Hdh^Q111^*/*Hdh^Q111^* cells expressing exogenous p65 subunit of NFkB (RelA/NFkB) compared to the control ST*Hdh^Q111^*/*Hdh^Q111^* cells; (**B**) Average luciferase activity of NFkB response element (NFkB-RE) in ST*Hdh^Q7^/Hdh^Q7^* cells and ST*Hdh^Q7^/Hdh^Q7^* cells expressing exogenous p65 subunit of NFkB (RelA/NFkB). Exogenous expression of p65 sub-unit of NFkB (RelA/NFkB) increased the luciferase activity significantly (n = 4, p = 0.022). Bars 3 and 4, from the left, represent changes in luciferase activity of NFkB-RE on treatment with 0.1 mM and 2 mM aspirin for 24 hours in ST*Hdh^Q7^/Hdh^Q7^* cells. Treatment with 2.0 mM aspirin reduced the luciferase activiy (n = 2, p = 0.014); (**C**) Fold increase in the expression of mature miR-146a detected by real time PCR using stem loop specific primers in p65 subunit of NFkB (RelA/NFkB) transfected ST*Hdh^Q7^/Hdh^Q7^* cells (n = 3, p = 0.036) and ST*Hdh^Q111^*/*Hdh^Q111^* cells (n = 3, p = 0.045). Expression of miR-17-5p was used as endogenous control. Expression of miR-146a was significantly higher in cells expressing p65 subunit of NFkB (RelA/NFkB); (**D**) Treatment with 2.0 mM aspirin decreased the expression of mature miR-146a in ST*Hdh^Q7^/Hdh^Q7^* cells (n = 2, p = 0.031). Error bars represent standard deviation s of more than 2 experiments and each experiment was done in duplicate. “*” represents statistical significance; * p≤0.05; ** p<0.01.

Given that RelA/NFkB regulates miR-146a expression [29] and above observations that both RelA/NFkB steady state level and activity are compromised in ST*Hdh^Q111^/Hdh^Q111^* cells, we tested whether exogenous expression of p65 sub-unit of NFkB (RelA/NFkB) could rescue the expression of miR-146a in these cells. Transfection of RelA/NFkB (denoted by p65 in **Figure 2A**) in ST*Hdh^Q7^/Hdh^Q7^* and ST*Hdh^Q111^/Hdh^Q111^* cells increased the expression of the gene as determined by western blot analysis. In such condition, NFkB activity as revealed by reporter luciferase assay, was also increased significantly (n = 4, p = 0.022) as shown in **Figure 2B**. Mature miR-146a expression was increased in such condition in both ST*Hdh^Q7^/Hdh^Q7^* cells (n = 3, p = 0.036) and ST*Hdh^Q111^/Hdh^Q111^* cells (n = 3, p = 0.045) as shown in **Figure 2C**. Even though miR-146a expression increased in ST*Hdh^Q111^/Hdh^Q111^* cells exogenously expressing RelA/NFkB in comparison to ST*Hdh^Q111^/Hdh^Q111^* cells with endogenous RelA/NFkB, it did not rescue up to the level observed in ST*Hdh^Q7^/Hdh^Q7^* cells. It is known that aspirin decreases NFkB activity [35]. We thus tested whether decreasing NFkB activity by aspirin could alter miR-146a expression. ST*Hdh^Q7^/Hdh^Q7^* cells treated with 2.0 mM aspirin for 24 hours decreased the basal NFkB activity significantly (n = 2, p = 0.014) (**Figure 2B**). In such condition, expression of miR-146a was significantly (n = 2, p = 0.031) reduced (**Figure 2D**). Taken together, we show that increasing the expression of RelA/NFkB in ST*Hdh^Q111^/Hdh^Q111^* cells increased miR-146a expression and reducing the expression of RelA/NFkB decreased miR-146a expression in ST*Hdh^Q7^/Hdh^Q7^* cells establishing that decreased RelA/NFkB expression could result in the decreased expression of miR-146a.

### Over expression of p53 in STHdh^Q111^/Hdh^Q111^ cells: role of miR-125b and miR-150

**Figure 3:**
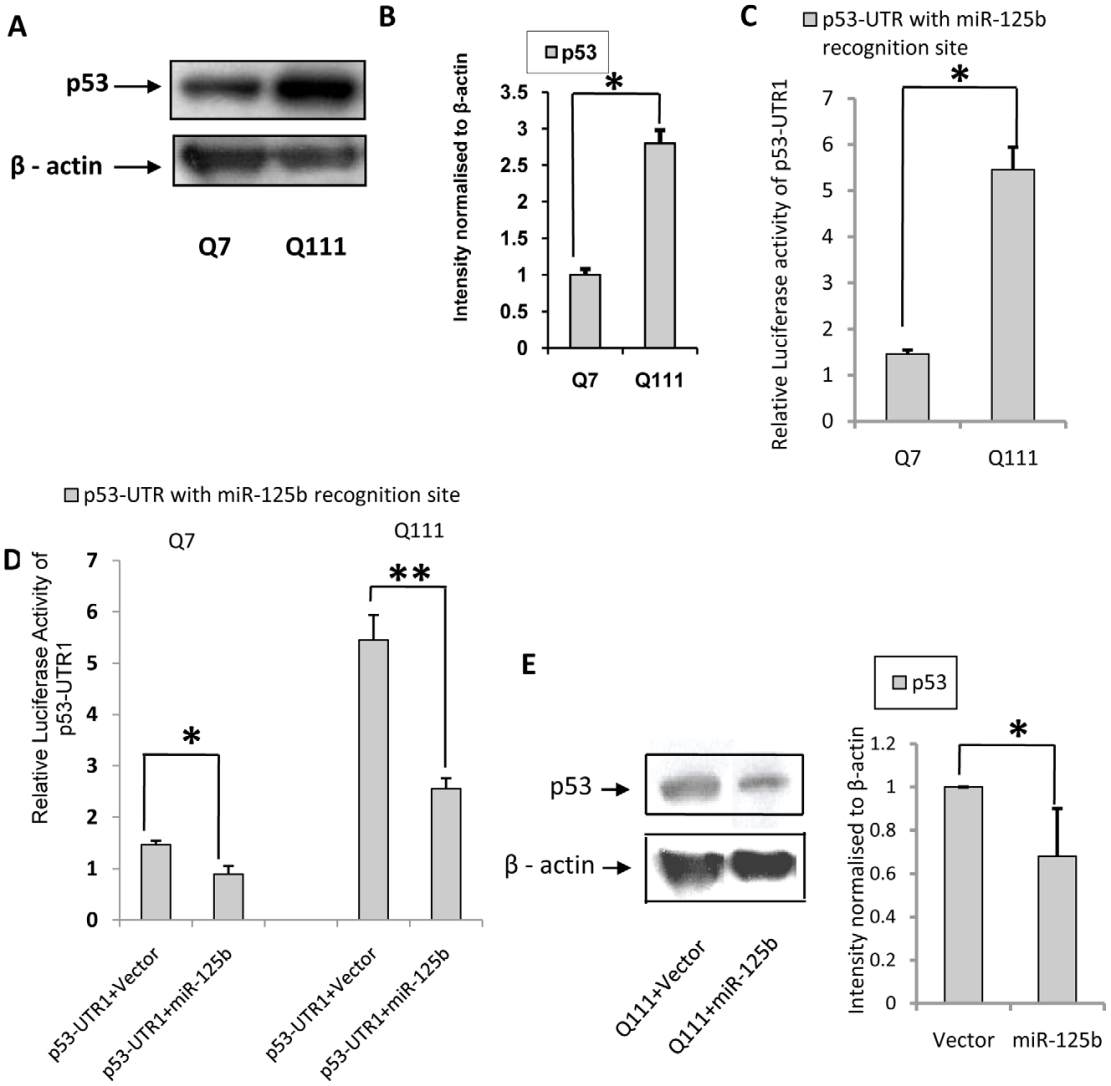
Endogenous expression of p53 in ST*Hdh^Q7^/Hdh^Q7^* and ST*Hdh^Q111^*/*Hdh^Q111^* cells: decreased miR-125b target p53. (**A**) Representative Western Blot showing increased p53 protein level in ST*Hdh^Q111^*/*Hdh^Q111^* cells compared to ST*Hdh^Q7^/Hdh^Q7^* cells; (**B**) Average integrated optical density (IOD) of p53 protein bands in *A*, normalized to β-actin level (n = 3, p = 0.024) in these cell lines. (**C**) Relative luciferase activity of cloned p53-3′UTR with miR-125b binding site (denoted by p53-UTR1) in ST*Hdh^Q111^*/*Hdh^Q111^* cells compared to ST*Hdh^Q7^/Hdh^Q7^* cells. Normalization of protein level between ST*Hdh^Q111^/Hdh^Q111^* cells and ST*Hdh^Q7^/Hdh^Q7^* cells was done by taking the ratio of RLU of cloned construct i.e. p53-UTR1 and empty vector pmiR. Relative luciferase activity of p53-UTR1 was found significantly higher (n = 3, p = 0.026) in ST*Hdh^Q111^/Hdh^Q111^* cells compared to ST*Hdh^Q7^/Hdh^Q7^* cells; (**D**) Reduced luciferase activity of p53-UTR1 co-transfected with pre-miR-125b in ST*Hdh^Q7^/Hdh^Q7^* cells (n = 3, p = 0.024) and ST*Hdh^Q111^/Hdh^Q111^* cells (n = 3, p = 0.0086) compared to those obtained in respective empty vector U61 tansfected cells; (**E**) Representative Western Blot showing reduction in p53 protein level in ST*Hdh^Q111^/Hdh^Q111^* cells 72 hours following transfection with pre-miR-125b compared to ST*Hdh^Q111^/Hdh^Q111^* cells transfected with empty vector U61, average IOD compared to β-actin (n = 3, p = 0.039) is shown in the adjacent bar diagram.

Expression of p53 is increased in ST*Hdh^Q111^/Hdh^Q111^* cells [32] as well as in various models of HD and post mortem HD brains. The exact mechanism for the increase of p53 protein in HD remains unknown. It has been shown by us that miR-125b is down regulated in ST*Hdh^Q111^/Hdh^Q111^* cells compared to the wild type cells [33]. Given that p53 is a validated target of miR-125b [28], we explored whether increased expression level of endogenous p53 in ST*Hdh^Q111^/Hdh^Q111^* cells could be due to decrease in the expression of miR-125b or any other miRNA down regulated in these cells [33]. We confirmed that the endogenous expression of p53 is increased in ST*Hdh^Q111^/Hdh^Q111^* cells (**Figures 3A and 3B**) compared to the wild type ST*Hdh^Q7^/Hdh^Q7^* cells. Luciferase activity of the reporter vector pmiR-Report with 150 bp (position 733–739) of the 3′-UTR of human p53 (p53-UTR1) containing miR-125b recognition site [28] was also significantly (n = 3, p = 0.026) increased in ST*Hdh^Q111^/Hdh^Q111^* cells compared to that observed in ST*Hdh^Q7^/Hdh^Q7^* cells (**Figure 3C**) indicating that down regulated miR-125b could target p53 and increase its expression. Further, over expression of pre-miR-125b that increased the expression of mature miR-125b significantly (data not shown) decreased reporter luciferase activity significantly in ST*Hdh^Q7^/Hdh^Q7^* cells (n = 3, p = 0.024) and ST*Hdh^Q111^/Hdh^Q111^* cells (n = 3, p = 0.0086) when co-expressed with p53-UTR1 (**Figure 3D** **)**. In addition, exogenous expression of miR-125b decreased the endogenous expression of p53 (n = 3, p = 0.039), shown in **Figure 3E**. Taken together, these results confirmed the earlier observation that p53 is one of the targets of miR-125b [28]. Thus, the increased expression level of endogenous p53 in ST*Hdh^Q111^/Hdh^Q111^* cells could be due to decreased expression of endogenous miR-125b.

**Figure 4:**
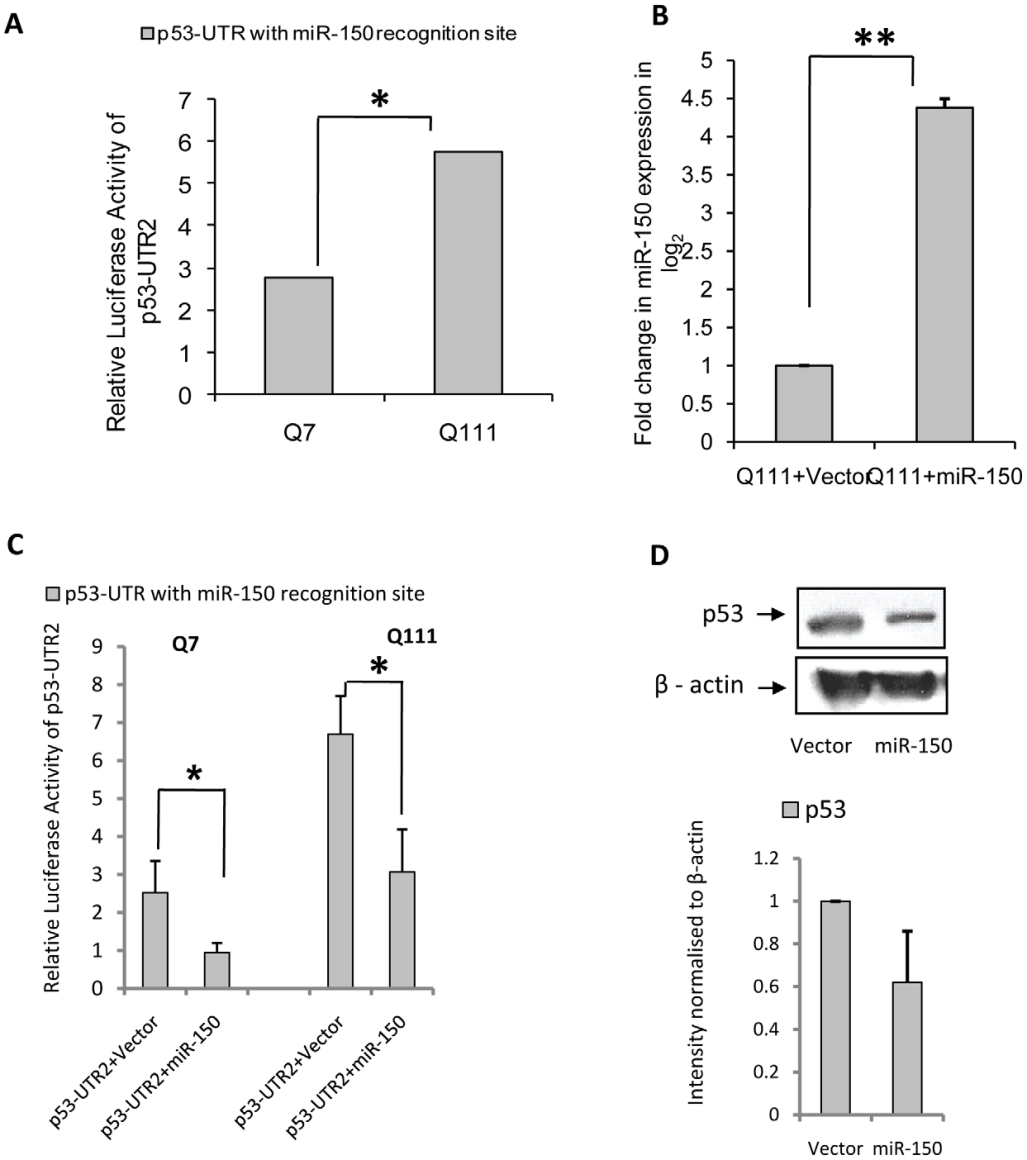
Reduced miR-150 also targets p53 in ST*Hdh^Q7^/Hdh^Q7^* and ST*Hdh^Q111^*/*Hdh^Q111^* cells. (**A**) Relative luciferase activity of cloned p53-3′UTR with miR-150 binding site (denoted by p53-UTR2) in ST*Hdh^Q111^*/*Hdh^Q111^* cells compared to ST*Hdh^Q7^/Hdh^Q7^* cells. Normalization of protein levels between the cells was done as described earlier. Relative luciferase activity of p53-UTR2 was found significantly higher (n = 3, p = 0.031) in ST*Hdh^Q111^/Hdh^Q111^* cells compared to ST*Hdh^Q7^/Hdh^Q7^* cells; (**B**) Fold increase (n = 3, p = 0.0056) in mature miR-150 expression detected by real time PCR using stem loop specific primers in ST*Hdh^Q111^/Hdh^Q111^* cells transfected with cloned pre-miR-150 compared to ST*Hdh^Q111^/Hdh^Q111^* cells transfected with empty vector U61, at 24 hours post transfection. Expression of miR-17-5p was used as endogenous control; (**C**) Reduced luciferase activity of p53-UTR2 co-transfected with pre-miR-150 in ST*Hdh^Q7^/Hdh^Q7^* cells (n = 3, p = 0.021) and ST*Hdh^Q111^/Hdh^Q111^* cells (n = 3, p = 0.04) compared to those obtained in respective empty vector U61 transfected cells; (**D**) Typical Western Blot showing reduction in p53 protein level in ST*Hdh^Q111^/Hdh^Q111^* cells 72 hours following transfection with pre-miR-150 compared to ST*Hdh^Q111^/Hdh^Q111^* cells transfected with empty vector U61. Average IOD compared to β-actin (n = 3, p = 0.043) is shown in the adjacent bar diagram.

We searched mirbase [36] (http://www.mirbase.org/) and observed that human p53 could also be targeted by miR-150, which is decreased in ST*Hdh^Q111^/Hdh^Q111^* cells [33]. We cloned 136 bp (position 234–256) of the 3′-UTR of p53 (p53-UTR2) containing the predicted recognition site of miR-150 in luciferase reporter vector as described above. We observed that the luciferase activity of the reporter with p53 3′-UTR containing the recognition site of miR-150, shown in **Figure S1 (A)**, was increased significantly (n = 3, p = 0.031) in ST*Hdh^Q111^/Hdh^Q111^* cells compared to that in ST*Hdh^Q7^/Hdh^Q7^* cells (**Figure 4A**). This result indicated that miR-150 could also target p53. We next cloned pre-miR-150 in pRNA-U61 vector. ST*Hdh^Q111^/Hdh^Q111^* cells transfected with this construct showed an increase in mature miR-150 levels as detected by Real Time PCR with stem loop specific primers. The result shown in **Figure 4B** was statistically significant (n = 3, p = 0.0056). Exogenous expression of cloned pre-miR-150 construct decreased the reporter luciferase activity of p53-UTR2 in both ST*Hdh^Q7^/Hdh^Q7^* (n = 3, p = 0.021) and ST*Hdh^Q111^/Hdh^Q111^* cells (n = 3, p = 0.040) as shown in **Figure 4C**. Moreover, over expression of pre-miR-150 decreased the endogenous expression of p53 in ST*Hdh^Q111^/Hdh^Q111^* cells (n = 3, p = 0.043) as shown by Western blot analysis (**Figure 4D**). These results indicate that p53 could be targeted by miR-150 as well.

**Figure 5:**
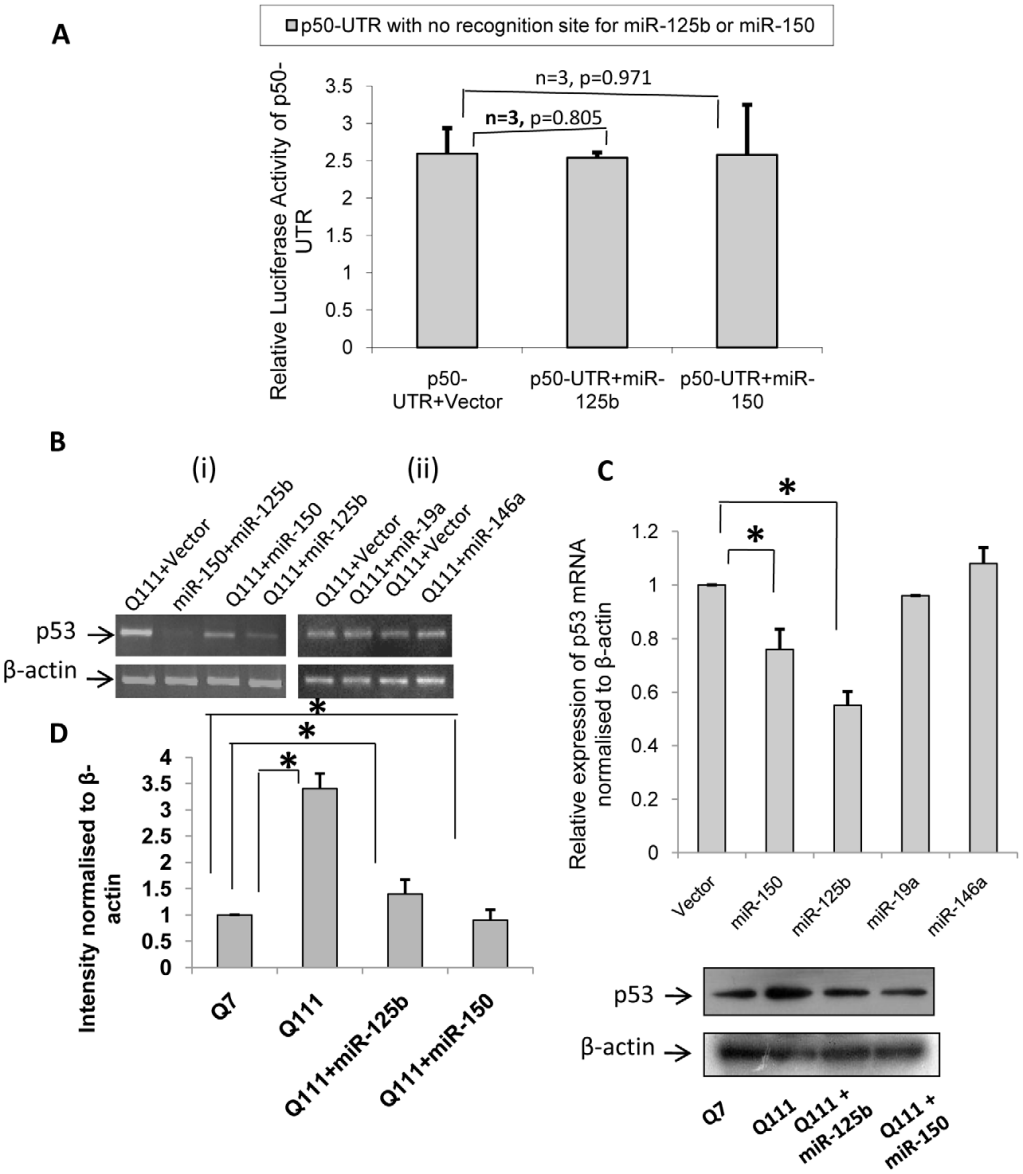
p53 is specifically targeted by miR-125b and miR-150 in HD cell model. (**A**) No change in relative luciferase activity of p50-3′UTR (bearing no predicted recognition site for miR-125b or miR-150) in cells co-transfected with pre- miR-125b and pre-miR-150 compared to cells co-transfected with empty vector U61; (**B**) RT-PCR showing (i) reduction in p53 mRNA in cells expressing exogenous pre-miR-150 and pre-miR-125b compared to cells expressing empty vector U61, (ii) no reduction in p53 mRNA in cells expressing exogenous pre-miR-19a and pre-miR-146a compared to cells expressing empty vector U61. (**C**) Average IOD showing relative expression of p53 mRNA in presence of over expressed miR-150 (n = 2, p = 0.021), miR-125b (n = 2, p = 0.029), miR-19a and miR-146a is given in the adjacent bar diagram (negative control). This indicates that p53 is specifically targeted by miR-125b and miR-150 in HD cell model. (**D**) Average IOD showing relative expression of p53 protein level in cell extracts prepared from ST*Hdh^Q7^/Hdh^Q7^*, ST*Hdh^Q111^/Hdh^Q111^* and in ST*Hdh^Q111^/Hdh^Q111^* cells transfected with miR-125b or miR-150. Immunoblot analysis show that the extent of p53 up regulation found in ST*Hdh^Q111^/Hdh^Q111^* cells when compared to ST*Hdh^Q7^/Hdh^Q7^* cells was reduced when ST*Hdh^Q111^/Hdh^Q111^* cells were transfected with miR-125b or miR-150.

As a negative control, we tested 213 bp (position 145–359 of the 3′ UTR) of p50 sub-unit of NFkB (also known as NFkB1) containing no predicted recognition sites for either miR-125b or miR-150 and did not observe any change in the luciferase activity significantly when the construct (p50-UTR) was co-transfected with cloned pre-miR-125b or pre-miR-150 in ST*Hdh^Q7^/Hdh^Q7^* cells (**Figure 5A**). This result showed that the decrease in the luciferase activity by exogenous expression of miR-150 was specific. Although endogenous p53 level was decreased by over expressing miR-125b or miR-150, there was no change in p53 level either in the presence of exogenous miR-19a or miR-146a (**Figure 5B and 5C**). Neither of these miRNAs has any predicted recognition site in the cloned 3′UTRs of p53 as revealed from miRbase. Taken together, these results show that p53 is specifically targeted by miR-125b and miR-150. Since the expressions of miR-125b and miR-150 were decreased in ST*Hdh^Q111^/Hdh^Q111^* cells compared to those obtained in ST*Hdh^Q7^/Hdh^Q7^* cells, we expressed these miRNAs in ST*Hdh^Q111^/Hdh^Q111^* cells and detected the endogenous expression of p53 as shown in **Figure 5D**. It is evident that exogenous expressions of both the miRNAs resulted in decreased expression of p53 in ST*Hdh^Q111^/Hdh^Q111^* cells. However, the level of expression did not reach exactly up to that of in ST*Hdh^Q7^/Hdh^Q7^* cells. Thus, decreased expressions of miR-125b and miR-150 in ST*Hdh^Q111^/Hdh^Q111^* cells could result in increased expression of p53.

### Role of p53 in the expression of miR-146a in STHdh^Q7^/Hdh^Q7^ and STHdh^Q111^/Hdh^Q111^ cells

**Figure 6:**
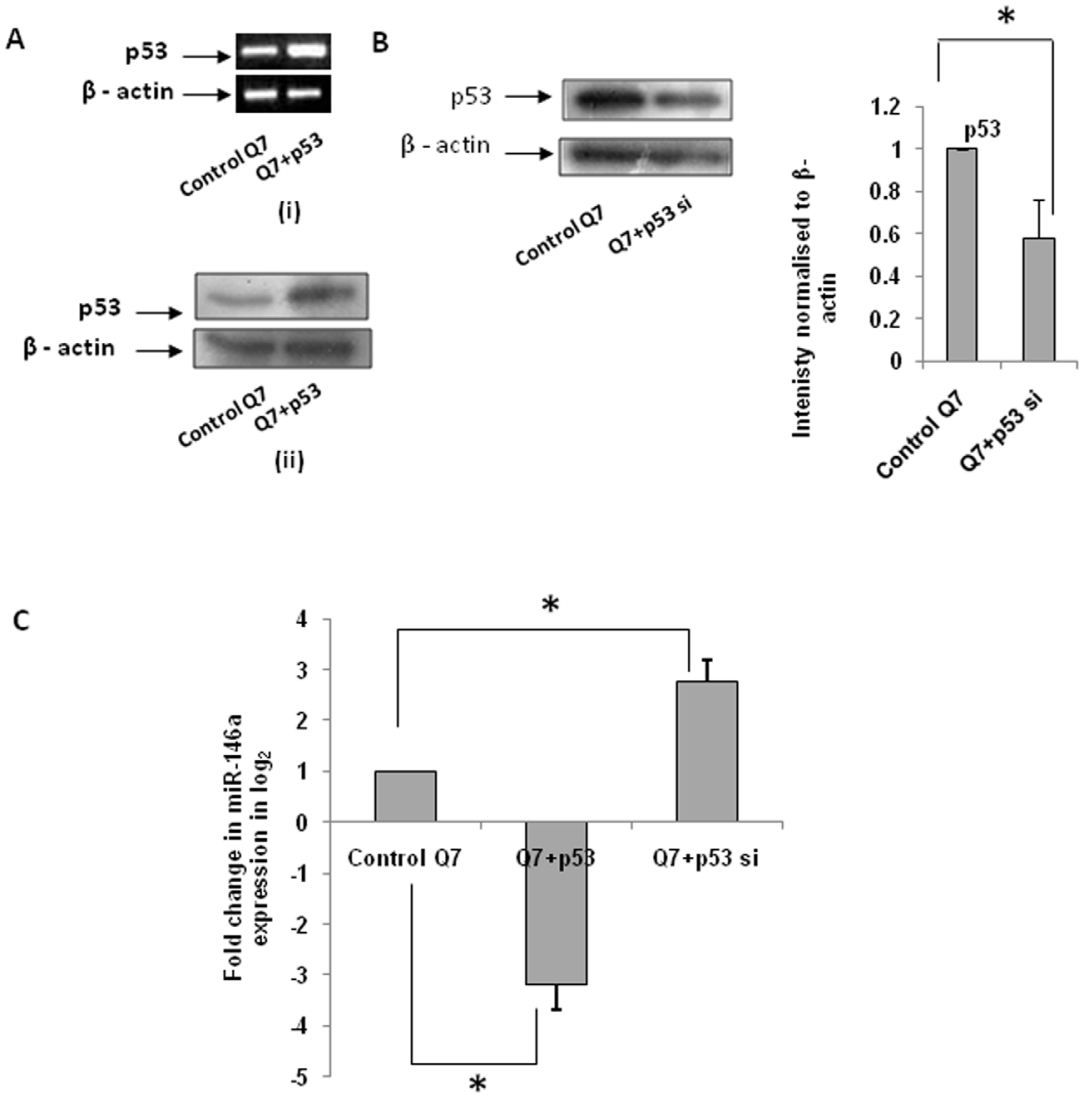
Role of p53 in the expression of miR-146a. (**A**) (i) RT-PCR showing p53 over expression upon transfection of exogenous p53-CFP in ST*Hdh^Q7^/Hdh^Q7^* cells compared to cells transfected with empty vector CFP and (ii) western blot showing p53 over expression at the protein level under similar conditions; (**B**) Representative Western Blot showing reduction in p53 protein level in ST*Hdh^Q7^/Hdh^Q7^* cells trasfected with pSuppressor plasmid containing p53 siRNA construct compared to ST*Hdh^Q7^/Hdh^Q7^* cells transfected with empty vector U61. Average IOD showing significant reduction in p53 protein level (n = 3, p = 0.041) compared to that of β-actin (control) is shown in the adjacent bar diagram; (**C**) Fold change in miR-146a expression in ST*Hdh^Q7^/Hdh^Q7^* cells in presence of over expressed p53 and reduced p53 levels respectively. miR-146a expression was significantly decreased (n = 3, p = 0.032) in ST*Hdh^Q7^/Hdh^Q7^* cells 72 hours post transfection with p53-CFP compared to empty vector CFP transfected cells and the expression was increased (n = 3, p = 0.029) in ST*Hdh^Q7^/Hdh^Q7^* cells compared to control ST*Hdh^Q7^/Hdh^Q7^* cells transfected with empty vector U61.

There are conflicting results regarding the functional interactions between RelA/NFkB and p53. Several reports show that p53 inhibits the transcriptional activity of RelA/NFkB [37]–[40] either by binding to the promoter sequences or by altering the interaction of NFkB with p53 and CBP. A different pathway has been identified where p53 enhances RelA/NFkB activity [41], [42]. These observations prompted us to find whether increased expression of p53 in ST*Hdh^Q111^/Hdh^Q111^* cells had any influence on the down regulation of miR-146a. We exogenously expressed p53 in ST*Hdh^Q7^/Hdh^Q7^* cells and knocked down p53 in ST*Hdh^Q111^/Hdh^Q111^* cells and also in ST*Hdh^Q7^/Hdh^Q7^* cells using validated siRNA commercially available from Imgenex Corporation. Exogenous expression of p53 in ST*Hdh^Q7^/Hdh^Q7^* cells was confirmed by RT-PCR **Figure 6A** **(i)** as well as by western blot analysis **Figure 6A** **(ii)** while down regulation of the protein by siRNA in ST*Hdh^Q7^/Hdh^Q7^* cells was confirmed by western blot analysis as shown in **Figure 6B**. Detection of the expression of mature miR-146a in these cells revealed that in the presence of exogenous p53, miR-146a was down regulated significantly (n = 3, p = 0.032) while as expected, knocking down p53 up regulated the expression of the miRNA (n = 3, p = 0.029) as shown in **Figure 6C**.

**Figure 7:**
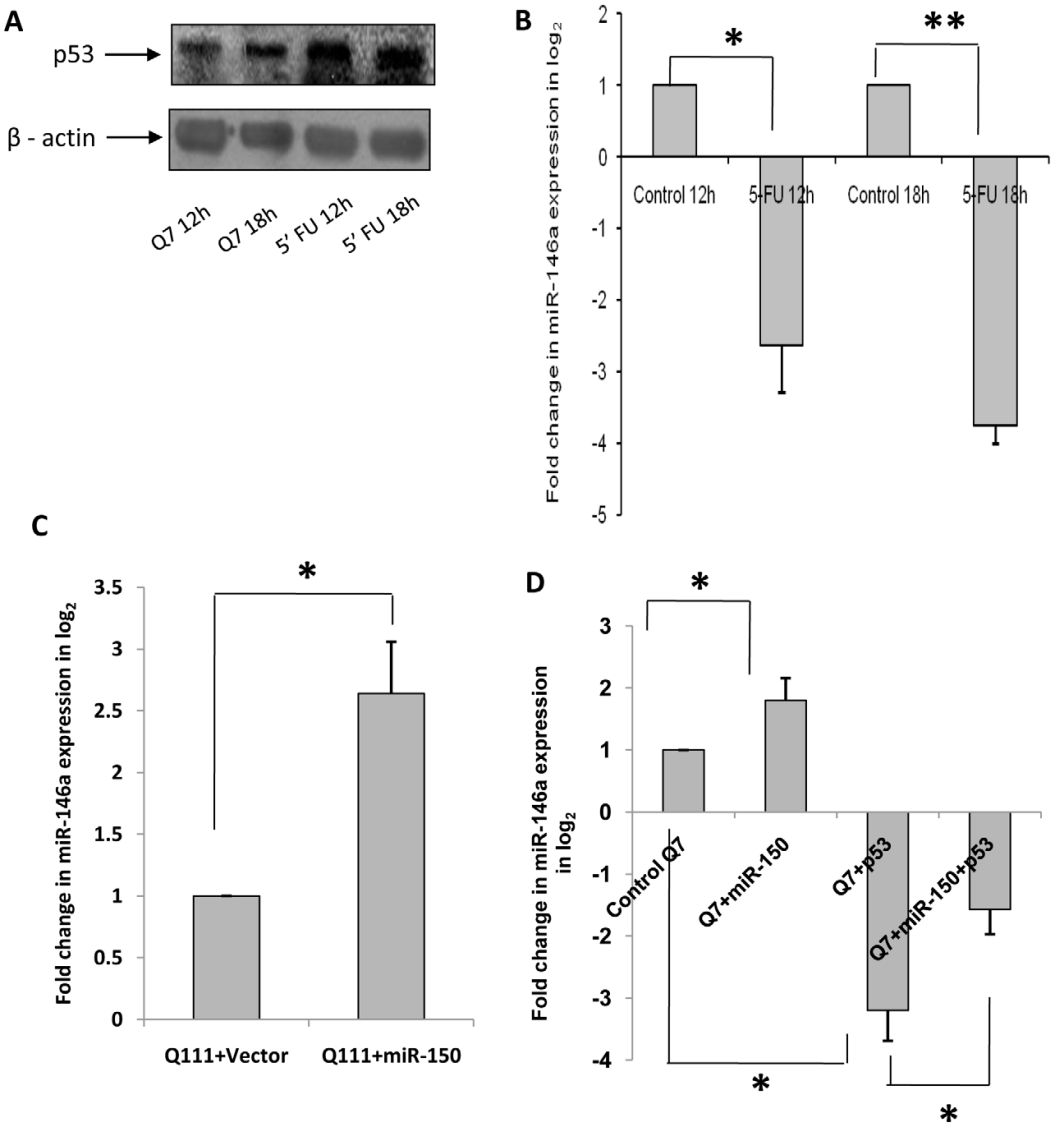
Stabilisation of p53 by 5-FU in ST*Hdh^Q7^/Hdh^Q7^* cells leads to decreased expression of miR-146a. (**A**) Typical Western Blot showing p53 stabilisation upon 5-FU treatment of ST*Hdh^Q7^/Hdh^Q7^* cells. ST*Hdh^Q7^/Hdh^Q7^* cells were treated with 10 µg/ml of 5-FU. Cells were harvested 12 hours and 18 hours successively post 5-FU treatment and immunoblotting with the cell extracts show an up regulation in p53 protein level in the treated cells compared to the untreated ones; (**B**) miR-146a expression was reduced significantly in ST*Hdh^Q7^/Hdh^Q7^* cells treated with 5-FU for 12 hours (n = 2, p = 0.038) and for 18 hours (n = 2, p = 0.0089) compared to the respective untreated cells; (**C**). Relative differences in miR-146a expression in presence of reduced p53 levels in ST*Hdh^Q111^/Hdh^Q111^* cells. miR-146a expression was increased significantly (n = 3, p = 0.039) in ST*Hdh^Q111^/Hdh^Q111^* cells 72 hours post transfection with pre-miR-150 compared to ST*Hdh^Q111^/Hdh^Q111^* cells transfected with empty vector U61. (**D**) Relative differences in miR-146a expression in endogenous ST*Hdh^Q7^/Hdh^Q7^* cells and in cells transfected respectively with pre-miR-150, p53 and pre-miR-150 and p53 alone. Mature miR-146a expression was increased significantly (n = 3, p = 0.03) in cells transfected with pre-miR-150 which has been shown to reduce p53. miR-146a expression was reduced in presence of p53 (n = 3, p = 0.032). However, the reduction was less in cells when p53 was co-transfected with pre-miR-150 (n = 3, p = 0.044).

To confirm further, we treated ST*Hdh^Q7^/Hdh^Q7^* cells with 5-Flurouracil (5-FU, 10 µg/ml for 12 h and 18 h), which is known to stabilize p53 protein [43]. In such condition, steady state level of p53 was increased (**Figure 7A**) and the expression of miR-146a was decreased significantly for both the time points as shown in **Figure 7B**. As we have shown above that miR-150 might target p53, we also expressed miR-150 in ST*Hdh^Q111^/Hdh^Q111^* cells and as expected, significant increase (n = 3, p = 0.039) in the expression of miR-146a was observed (**Figure 7C**), possibly due to down regulation of p53 by miR-150. Similar increase in miR-146a expression was observed when ST*Hdh^Q7^/Hdh^Q7^* cells were transfected with miR-150. However, when p53 was co-transfected with miR-150, no increase in the expression of miR-146a was observed. This result shows that decrease in p53 expression by miR-150 could be compensated here by the exogenous expression of p53 which does not have the 3′-UTR region bearing the target site of miR-150. Indeed miR-146a was down regulated when p53 was co-transfected with miR-150. However, the extent of decrease was less compared to when ST*Hdh^Q7^/Hdh^Q7^* cells were transfected with p53 alone as shown in **Figure 7D**. These results showed that in our experimental conditions, p53 directly or indirectly regulates the expression of miR-146a.

### Regulation of p65 subunit of NFkB (RelA/NFkB) expression and activity by exogenous p53

**Figure 8:**
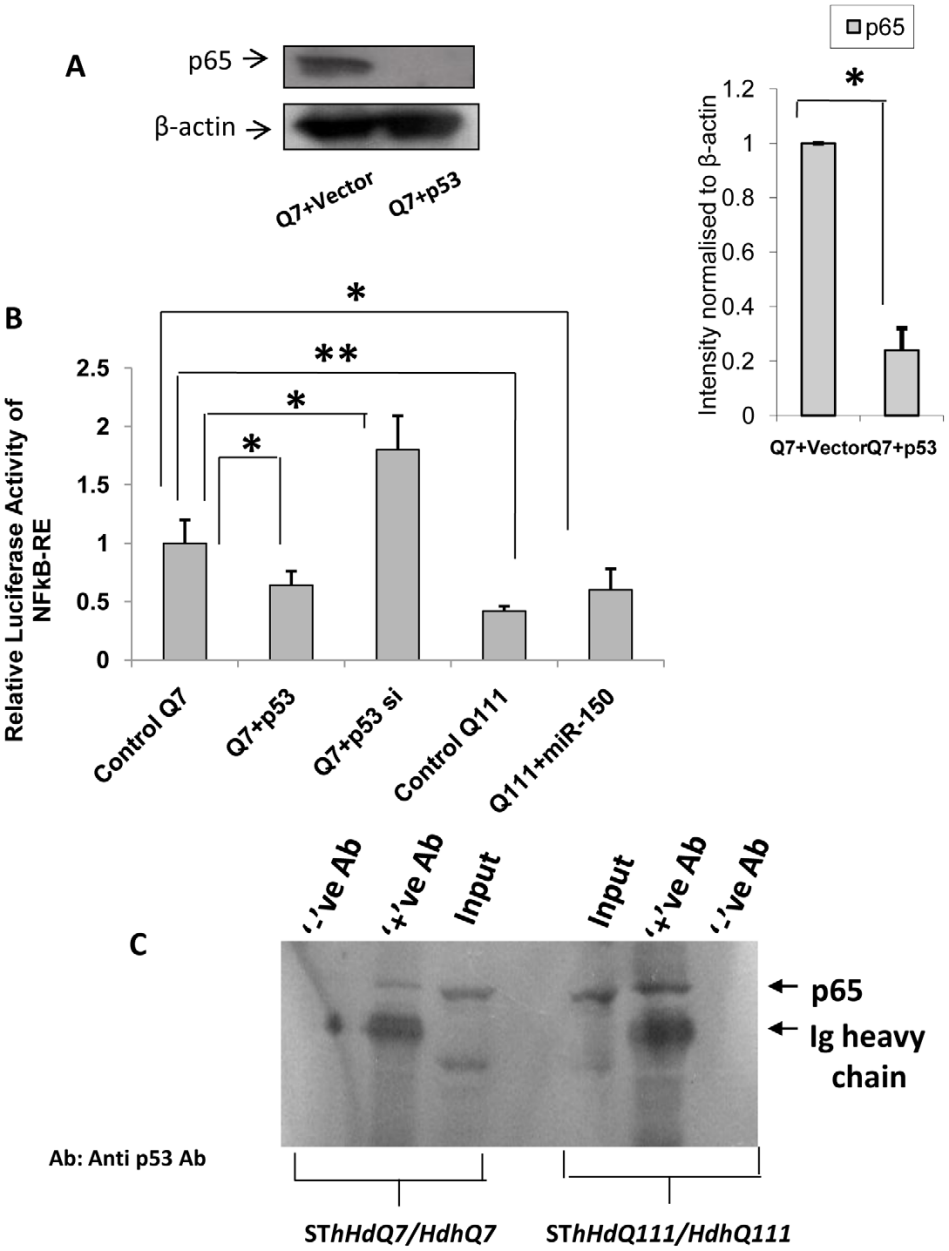
Regulation of RelA/NFkB expression and activity by p53. (**A**) Typical experiment by Western Blot showing decrease in NF-kB (p65) i.e. RelA/NFkB expression upon over expression of p53 in ST*Hdh^Q7^/Hdh^Q7^* cells. RelA/NFkB expression was decreased significantly (n = 3, p = 0.0041) in ST*Hdh^Q7^/Hdh^Q7^* cells 48 hours post transfection with p53-CFP compared to ST*Hdh^Q7^/Hdh^Q7^* cells transfected with empty vector CFP. Average IOD obtained by comparing with β-actin is shown in the adjacent bar diagram; (**B**) Decrease in luciferase activity (n = 3, p = 0.0214) of NFkB-RE in ST*Hdh^Q7^/Hdh^Q7^* cells 48 hours post transfection with p53-CFP compared to ST*Hdh^Q7^/Hdh^Q7^* cells transfected with empty vector CFP and increase in luciferase activity (n = 3, p = 0.032) of NFkB-RE in ST*Hdh^Q7^/Hdh^Q7^* cells 48 hours post transfection with p53 si compared to control ST*Hdh^Q7^/Hdh^Q7^* cells; increase in luciferase activity (n = 4, p = 0.0482) of NFkB-RE in ST*Hdh^Q111^/Hdh^Q111^* cells 48 hours post transfection with pre-miR-150 compared with that obtained in ST*Hdh^Q111^/Hdh^Q111^* cells transfected with empty vector U61. Relative luciferase activity of NFkB-RE obtained in control Q7 was taken as 1; (**C**) Co-immunoprecipitation analysis showing in vivo interaction of p53 with p65 sub-unit of NF-kB (RelA/NFkB) in wild type ST*Hdh^Q7^/Hdh^Q7^* cells and mutant ST*Hdh^Q111^/Hdh^Q111^* cells. Cell extracts prepared from ST*Hdh^Q7^/Hdh^Q7^* cells and ST*Hdh^Q111^/Hdh^Q111^* cells were immunoprecipitated by anti-p53 antibody coupled to agarose-protein G beads. Western blotting the p53 immunoprecipitated complex with anti-p65 antibody reveals such interaction as evident in lanes 2 and 6 denoted by ‘+’ ve Ab.

It has been mentioned in the preceding section that there are conflicting results regarding the functional interactions between RelA/NFkB and p53. This conflicting result could arise from the dependence of cellular needs in different conditions of growth as well as for different types of cells. Depending on cellular needs, p53 may modulate NFkB activity differently. We tested whether p53 directly or indirectly regulates NFkB (RelA/NFkB) expression and activity. Exogenous expression of p53 significantly (n = 3, p = 0.041) reduced the steady state level of RelA/NFkB) in ST*Hdh^Q7^/Hdh^Q7^* cells (**Figure 8A**). Exogenous expression of p53 in ST*Hdh^Q7^/Hdh^Q7^* cells significantly decreased (n = 3, p = 0.021) the activity of NFkB whereas knocking down p53 by siRNA led to an increase in NFkB activity (n = 3, p = 0.032) in ST*Hdh^Q7^/Hdh^Q7^* cells. Reduction of p53 expression in ST*Hdh^Q111^/Hdh^Q111^* cells by expressing miR-150 that targets p53, significantly increased NFkB activity (n = 4, p = 0.0482) as detected by luciferase reporter assay (**Figure 8B**). Similar results were also obtained in HeLa cells expressing exogenous p53 and by knocking down p53 by siRNA (data not shown). Thus, in the presence of excess p53, RelA/NFkB expression and activity are reduced. This result showed that increased p53 in ST*Hdh^Q111^/Hdh^Q111^* cells might reduce NFkB activity. Besides, there are evidence of physical interaction between (RelA/NFkB) and p53 [37]. By co-immuno precipitation analysis, we confirmed such interaction in both ST*Hdh^Q7^/Hdh^Q7^* and ST*Hdh^Q111^/Hdh^Q111^* cells (**Figure 8C**). However, it remains unknown how p53 negatively regulates RelA/NFkB expression.

### Poly Q aggregates cause alterations in the expressions of protein coding genes and miRNAs and removal of aggregates by chaperones rescue such changes

**Figure 9:**
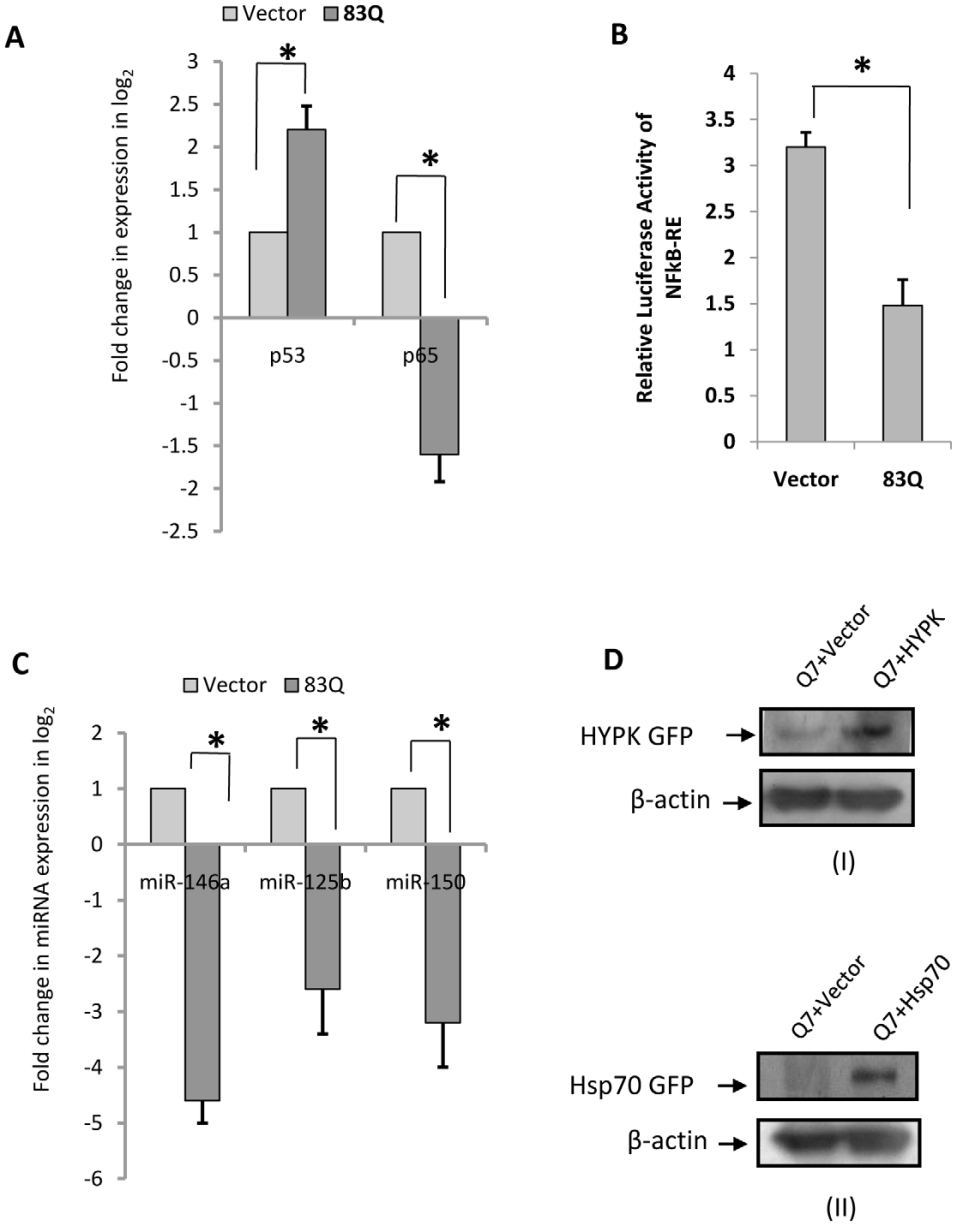
Poly Q aggregates alter the expressions of miR-125b, miR-150, p53, RelA/NFkB and miR-146a. (**A**) Increase in p53 expression (n = 3, p = 0.028) and decrease in RelA/NFkB expression (n = 3, p = 0.031) in 83Q DsRed transfected ST*Hdh^Q7^/Hdh^Q7^* cells compared to ST*Hdh^Q7^/Hdh^Q7^* cells transfected with empty vector DsRed. (**B**) Decrease in relative luciferase activity of NFkB-RE upon 83Q DsRed transfection in ST*Hdh^Q7^/Hdh^Q7^* cells. Average luciferase activity of NFkB-RE was significantly decreased (n = 6, p = 0.0334) in ST*Hdh^Q7^/Hdh^Q7^* cells 48 hours post transfection with 83Q DsRed compared to ST*Hdh^Q7^/Hdh^Q7^* cells transfected with empty vector DsRed. (**C**) Similarly, miR-146a expression (n = 4, p = 0.011), miR-125b expression (n = 2, p = 0.048) and miR-150 expressions (n = 2, p = 0.039) were decreased significantly in ST*Hdh^Q7^/Hdh^Q7^* cells 48 hours post transfection with 83Q DsRed compared to ST*Hdh^Q7^/Hdh^Q7^* cells 48 hours post transfection with empty vector DsRed. miR-17-5p was used as endogenous control to calculate fold change in each case; (**D**) Immunoblotting with cell extracts prepared from HYPK-GFP transfected ST*Hdh^Q7^/Hdh^Q7^* cells and Hsp70-GFP transfected ST*Hdh^Q7^/Hdh^Q7^* cells showed increase in the expression of HYPK (in panel I probed by anti-HYPK antibody) and Hsp70 (in panel II probed by anti-GFP antibody) respectively compared to those obtained in ST*Hdh^Q7^/Hdh^Q7^* cells transfected with empty vector GFP-C1.

Formation of mutant HTT aggregates is the hallmark of HD and has been shown in several studies using cell [44] and animal models of HD as well as in the post mortem brains of HD patients. Recently, we have shown that HYPK, an interacting partner of HTT, possesses chaperone like activity and reduces mutant HTT aggregates and toxicity [45]. Besides, other chaperones including Hsp70 reduce mutant HTT aggregates [46]. Expression of DsRed tagged N-terminal HTT with 83Q coded by exon1 of *HTT* gene in ST*Hdh^Q7^/Hdh^Q7^* cells increased mutant HTT aggregates and in the presence of exogenous HYPK such aggregates are reduced (data not shown), similar to that which has been published earlier by us in other neuronal cells [44]. Expression of p53 was increased and RelA/NFkB expression was decreased (**Figure 9A**) in the presence of aggregates as revealed by RT-PCR. In such conditions, NFkB activity as determined by reporter luciferase assay was significantly (n = 6, p = 0.0334) decreased (**Figure 9B**). Expression of miR-146a was also significantly (n = 4, p = 0.011) decreased along with the expression of miR-125b and miR-150 as shown in **Figure 9C**, similar to that which has been shown in ST*Hdh^Q111^/Hdh^Q111^* cells [33]. HYPK-GFP and Hsp70-GFP were transfected into the cells which increased the level of HYPK and Hsp70 respectively as shown by Western blot analysis in **Figure 9D**.

**Figure 10:**
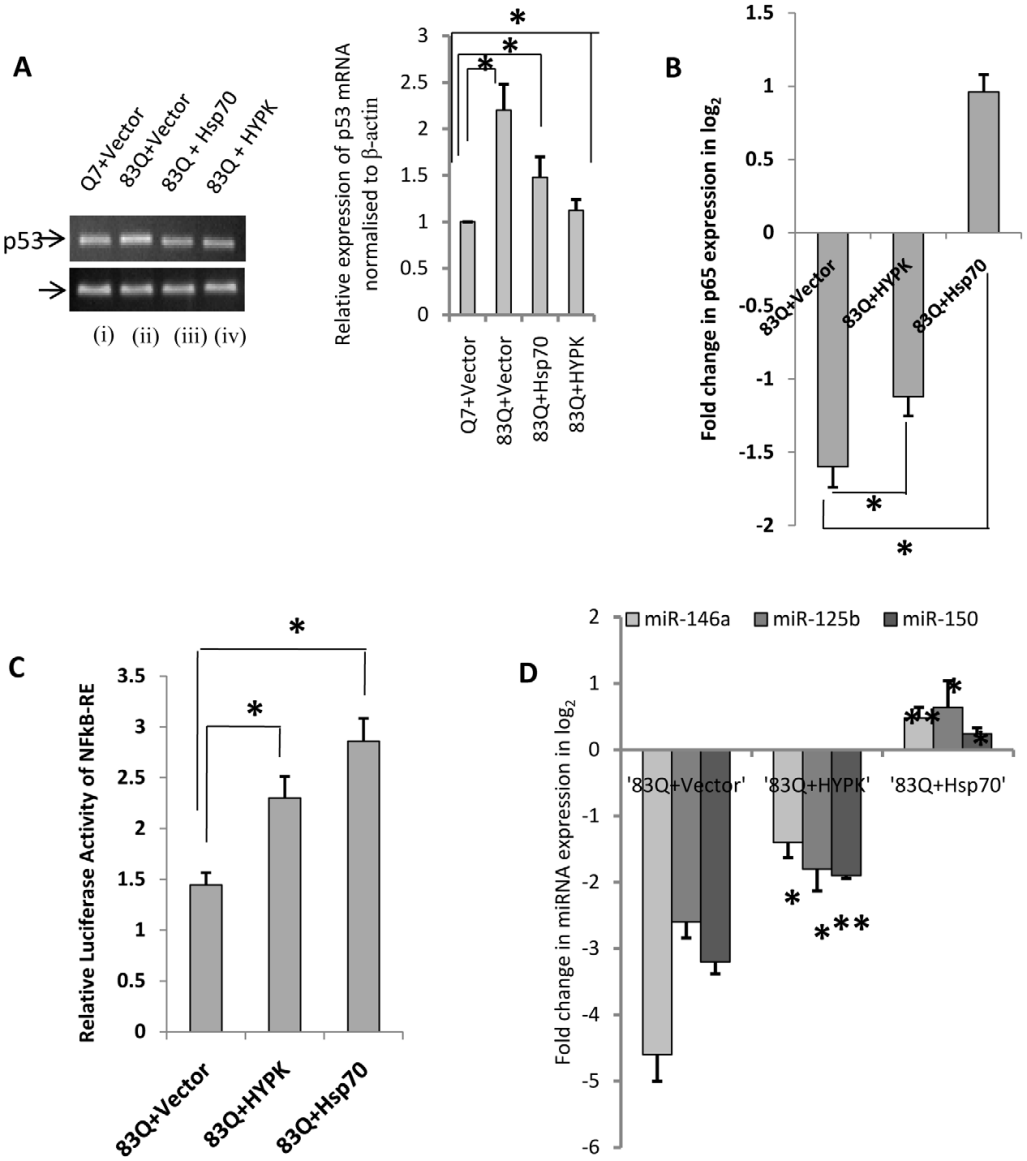
Removal of aggregates by chaperones rescue expression pattern of micoRNAs, p53 and RelA/NFkB. (**A**) RT-PCR showing increase in p53 expression in ST*Hdh^Q7^/Hdh^Q7^* cells co-transfected with 83Q DsRed and empty vector GFP-C1 (lane ii, n = 3, p = 0.028) and decrease in p53 expression in ST*Hdh^Q7^/Hdh^Q7^* cells co-transfected with 83Q DsRed and Hsp70-GFP (lane iii, n = 3, p = 0.025) and 83Q DsRed and HYPK-GFP (lane iv, n = 3, p = 0.019) compared to control ST*Hdh^Q7^/Hdh^Q7^* cells (lane i). Average IOD showing relative expression of p53 mRNA in each case is given in the adjacent bar diagram. (**B**) Changes in p65 (RelA/NFkB) expression in ST*Hdh^Q7^/Hdh^Q7^* cells co-transfected with 83Q DsRed and HYPK-GFP and 83Q DsRed and Hsp70-GFP compared to ST*Hdh^Q7^/Hdh^Q7^* cells co-transfected with 83Q DsRed and empty vector GFP-C1. The increase in RelA/NFkB expression was significant in the presence of HYPK-GFP (n = 6, p = 0.028) and also in the presence of Hsp70-GFP (n = 6, p = 0.022). (**C**) Revival of luciferase activity of NFkB-RE in presence of chaperones in 83Q DsRed transfected ST*Hdh^Q7^/Hdh^Q7^* cells. Luciferase activity of NFkB-RE in ST*Hdh^Q7^/Hdh^Q7^* cells co-transfected with 83Q DsRed and HYPK-GFP was significantly increased (n = 6, p = 0.031) when compared to that obtained in ST*Hdh^Q7^/Hdh^Q7^* cells transfected with 83Q DsRed and empty vector GFP-C1. Similar increase (n = 6, p = 0.029) in relative luciferase activity of NFkB-RE was observed in ST*Hdh^Q7^/Hdh^Q7^* cells 48 hours post transfection with 83Q DsRed and Hsp70-GFP; (**D**) Similarly, miR-146a expression (n = 3, p = 0.033), miR-125b expression and miR-150 expression were significantly increased in ST*Hdh^Q7^/Hdh^Q7^* cells 48 hours post transfection with 83Q DsRed and HYPK-GFP compared to ST*Hdh^Q7^/Hdh^Q7^* cells transfected with 83Q DsRed and empty vector GFP-C1. Such increase in miR-146a expression (n = 3, p = 0.0079), miR-125b expression and miR-150 expression were also observed with ST*Hdh^Q7^/Hdh^Q7^* cells 48 hours post transfection with 83Q DsRed and Hsp70-GFP. miR-17-5p was used as endogenous control to calculate fold change in each case.

Interestingly, when 83Q was co-transfected with HYPK-GFP or Hsp70-GFP in ST*Hdh^Q7^/Hdh^Q7^* cells, p53 expression was decreased and RelA/NFkB expression was recovered in comparison to that obtained in ST*Hdh^Q7^/Hdh^Q7^* cells expressing only mutant HTT exon1 (**Figure 10A and 10B**). NFkB activity was also significantly increased in such conditions in presence of HYPK-GFP (n = 6, p = 0.031) and Hsp70-GFP (n = 6, p = 0.029) in 83Q-DsRed transfected ST*Hdh^Q7^/Hdh^Q7^* cells (**Figure 10C**). Moreover, removal of aggregates by HYPK and Hsp70 also rescued the expression of miR-146a, miR-125b and miR-150 (**Figure 10D**). It is to be noted that the ability to recover the expressions of miR-125b, miR-146a and miR-150 by Hsp70 was higher compared to that obtained with HYPK, reasons remaining unknown. This result shows that mutant HTT aggregates directly or indirectly increased p53 expression, reduced RelA/NFkB expression and activity and also reduced miR-146a, miR-125b and miR-150 expressions.

### Expressions of p53, RelA/NFkB, miR-125b, miR-146a and miR-150 in striatal region of the brains of R6/2 mice

**Figure 11:**
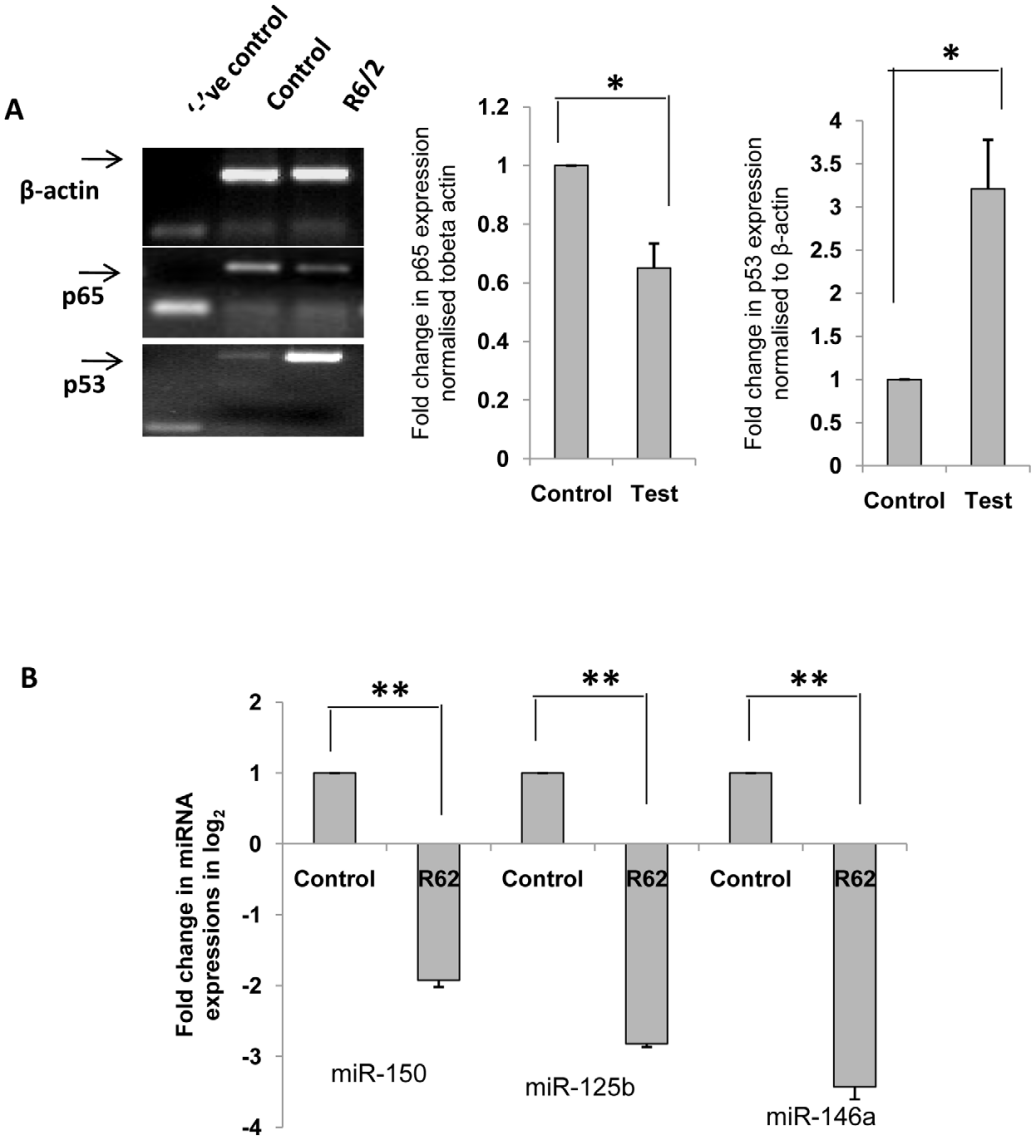
Expression pattern of p53, RelA/NFkB and microRNAs in striatal regions of the brains of R6/2 mice. (**A**) RT-PCR showing expression levels of β-actin, p65 sub-unit of NFkB (RelA/NFkB) and p53 in striatal regions of the brains of R6/2 mice and their age matched controls. Integrated optical density showing relative expression of p65 sub-unit of NFkB (RelA/NFkB) and p53 in the tissues normalized to β-actin are given in the adjacent bar diagrams. RelA/NFkB expression was found to be lesser (n = 3, p = 0.041) and p53 expression greater (n = 3, p = 0.05) in striatum of R6/2 mice when compared to their age matched controls. (**B**) Real time PCR analysis showing significant decrease in mature miR-150 expression (n = 3, p = 0.01), mature miR-125b expression (n = 3, p = 0.002) and mature miR-146a expression (n = 3, p = 0.008) in striatum of R6/2 mice.

Transgenic mice (R6/2 strain), an animal model of HD [47] has been widely used by many investigators. Total RNA was isolated from paraffinised tissue sections of these R6/2 mice and their age-matched controls as described in the materials and methods section. cDNA was prepared using random hexamer to determine the expressions of p53 and RelA/NFkB in the striatal tissues. Result obtained by RT-PCR revealed that expression of p53 was increased significantly (n = 3, p = 0.05), while the expression of RelA/NFkB was decreased significantly (n = 3, p = 0.04) compared to control (**Figure 11A**). As described above similar result was obtained in cell models of HD (**Figures 1A** **,** **3A** and **9A**). Besides, cDNA prepared using stem-loop specific primers for mature miR-125b, miR-146a and miR-150 also revealed a decrease in the expressions of these miRNAs (n = 3, p<0.01), similar to that obtained in ST*Hdh^Q111^/Hdh^Q111^* cells [33]. This result shown in **Figure 11B** reveals that the alterations in the expressions of p53, RelA/NFkB, miR-125b, miR-146a and miR-150 might be involved in the pathogenesis of HD.

In order to see whether miR-146a, miR-125b and miR-150 were specifically down regulated than others in striatal region of the brains of R6/2 mice, we determined the expression levels of additional ten miRNAs in the mouse model and compared the results with that obtained earlier by us in HD cell model [33]. The results given in **Table S1** show that expressions of miR-100, miR-125b, miR-135a, miR-138, miR-150, miR-146a, miR-221 which were decreased in HD cell model [33] were also decreased in R6/2 mice and the expressions of miR-127-3p and miR-214 were increased in both ST*Hdh^Q111^/Hdh^Q111^* cells [33] and the R6/2 mouse model. miR-145, miR-148a, miR-190 and miR-335 however showed different expression pattern in R6/2 mice and HD cell model [33]. Thus, out of the thirteen miRNAs whose expressions have been studied, expressions of eight miRNAs including miR-146a, miR-125b and miR-150 were decreased, expressions of two miRNAs (viz., miR-127 and miR-214) were increased and expressions of three miRNAs (viz., miR-145, miR-190 and miR-335) remained unchanged in R6/2 mice. Earlier observations by others showed miR-138, miR-218 and miR-222 to be down regulated in HD mouse models [30], [48] which had also been confirmed by us in HD cell model [33].

We have already shown in the earlier section that Poly Q aggregates cause decrease in the expressions of miR-146a, miR-125b and miR-150 and removal of aggregates by chaperones rescue such changes. To address the specificity of such alteration of miR-146a, miR-125b and miR-150 in the presence of poly Q aggregates, mutated exon1 of HTT gene that translated to N-terminal HTT with 83 Q was exogenously expressed in four different cell lines viz., Neuro2A (mouse neuroblastoma cell line), ST*Hdh^Q7^/Hdh^Q7^* (mouse sriatal cells having two copies of full length HTT with 7Q), SH-SY5Y (human neuroblastoma cell line) and HeLa cells (human cells derived from cervical tumours) and the expression of twenty two miRNAs were studied in these cell models (**Table S1**). Similar cell models of HD had earlier been shown by us [44], [45] and others. Of these twenty two miRNAs taken for study, eleven miRNAs were earlier found to be up regulated and eleven miRNAs were found to be down regulated by us in HD cell model [33]. Among the up regulated miRNAs in ST*Hdh^Q111^/Hdh^Q111^* cells, expressions of miR-214, miR-299 and miR-335 were also up regulated in three of the four cell models and expression of miR-199a was increased in two cell models. Expression of miR-148a, which was increased in ST*Hdh^Q111^/Hdh^Q111^* cells, was however decreased in all the four cell models. Among the down regulated miRNAs, expression of miR-146a was decreased in all the four cell models whereas expression of miR-125b and miR-150 were decreased in three of the four cell models excepting Neuro2A where expressions of those were up regulated. Expressions of miR-100 and miR-135b were also decreased in two of the four cell models. The results shown in **Table S1** suggests that although there is a heterogeneity in the expressions of miRNAs in different cell lines exogenously expressing mutated exon1 of HTT, miR-146a, miR-125b and miR-150 were preferentially decreased than others in the presence of poly Q aggregates.

### A probable model showing the involvement of NFkB (RelA), p53 and miRNAs in the regulation of cell death in HD pathogenesis

The model shows that mutant HTT modulates the expressions of both p53 and RelA/NFkB, NFkB activity and decreases miR-146a, miR-125b and miR-150 expressions. In the presence of mutant HTT aggregates, miR-125b and miR-150 expressions decrease leading to an increased level of p53. The elevated p53 then in turn, further increases mutant HTT aggregates and decreases RelA/NFkB expression, NFkB activity and miR-146a expression.

## Discussion

In this study, we present evidences to show that (i) in ST*Hdh^Q111^/Hdh^Q111^* cells decreased expression of miR-146a is mediated through decreased expression and activity of RelA/NFkB, (ii) increased expression of p53 in the same cells could be due to decreased expression of miR-125b and miR-150, (iii) p53 and RelA/NFkB regulate the expression of miR-146a and (iv) neuronal cells expressing N-terminal HTT with 83Q coded by exon1 exhibit decreased miR-125b and miR-150 expressions, increased p53 expression and reduced RelA/NFkB expression and activity and miR-146a expression. Such changes could be rescued by the expression of HYPK and Hsp70. Besides, we also show that expressions of miR-125b, miR-146a, miR-150 and RelA/NFkB were decreased while the expression of p53 was increased in striatal tissues of R6/2 mice models of HD.

Transcription factor RelA/NFkB is known to regulate the expression of miR-146a by binding to the upstream sequences [29]. RelA/NFkB dependent increase in the expression of miR-146a is shown earlier by several investigators in Alzheimer's disease (AD), viral infection, epilepsy and prion disease [49]–[52]. Increased expression of miR-146a results in the decreased expression of complement factor H (CFH) in AD and Herpes simplex virus type1 [49], [50]. Even though elevated expression of miR-146a is reported in epilepsy and scrapie, no targets of the miRNA are reported. The reason for the difference in the expression of the miRNA in AD and HD remains unknown. Here, we show that the steady state level and activity of RelA/NFkB are reduced in ST*Hdh^Q111^/Hdh^Q111^* cells compared to those in ST*Hdh^Q7^/Hdh^Q7^* cells (**Figures 1A–1D**). Exogenous expression of RelA/NFkB restores NFkB activity as well as the expression of miR-146a (**Figures 2A, 2B and 2C**). Reducing the activity of NFkB by treatment with aspirin [35] also compromised miR-146a expression (**Figure 2D**). Taken together, the down regulation of miR-146a in ST*Hdh^Q111^/Hdh^Q111^* cells seen earlier [33] could be due to lower steady state level of RelA/NFkB in these cells. We then confirmed the earlier observation that the level of p53 is increased in ST*Hdh^Q111^/Hdh^Q111^* cells and also in other cell and animal models of HD [10], [11], [32]. However, the mechanism of such increased level of p53 was not known. Since p53 is a known target of miR-125b [28] and the expression of miR-125b is down regulated in ST*Hdh^Q111^/Hdh^Q111^* cells [33], we tested the hypothesis that increased expression of p53 in these cells could be due to decreased level of miR-125b. Increased reporter luciferase activity of human p53 3′-UTR (718 to 742) containing miR-125b recognition site viz., p53-UTR1 in ST*Hdh^Q111^/Hdh^Q111^* cells compared to that in ST*Hdh^Q7^/Hdh^Q7^* cells (**Figure 3C**) and decreased luciferase activity of the same in presence of exogenous miR-125b indicated that miR-125b could target p53 (**Figure 3D**). Using the prediction tool RNAhybrid [53] http://bibiserv.techfak.uni-bielefeld.de/rnahybrid/), we observed that mouse p53 (*Trp53*) could also be targeted by miR-125b at 3′-UTR position 413–435 as shown in **Figure S1 (B)**. Exogenous expression of miR-125b decreased the endogenous level of p53 in ST*Hdh^Q111^/Hdh^Q111^* cells (**Figure 3E**). These results confirmed that in ST*Hdh^Q111^/Hdh^Q111^* cells, increased p53 level could be mediated by decreased expression of miR-125b. Significant decrease of mature miR-150 was detected in ST*Hdh^Q111^*/^Q111^ cells compared to that obtained in ST*Hdh^Q7^/Hdh^Q7^* cells [33] and also in neuronal cells expressing mutated exon1 of the *HTT* gene as well as in the post mortem brain of HD mice R6/2. We confirmed the prediction that human p53 could be a target of miR-150 at human p53 3′-UTR position 234–256. Mouse p53 (*Trp53*) could also be a target of miR-150 at 3′ UTR position 260–287 as shown in **Figure S1 (C)** using RNAhybrid [53]. Increased expression of luciferase reporter with predicted recognition site of miR-150 at the 3′-UTR of human p53 (p53-UTR2) in ST*Hdh^Q111^/Hdh^Q111^* cells in comparison to that in ST*Hdh^Q7^/Hdh^Q7^* cells was observed (**Figure 4A**). In the presence of exogenous miR-150, decreased expression of the same luciferase reporter in ST*Hdh^Q7^/Hdh^Q7^* cells (**Figure 4C**) and reduction of endogenous p53 in ST*Hdh^Q111^/Hdh^Q111^* cells (**Figure 4D**) were also observed. These results show that miR-150 also targets p53. However, 213 bp (145–359) of the 3′ UTR of NFkB1 (p50-UTR) containing no predicted binding site for either miR-125b or miR-150 showed no change in its luciferase activity (negative control) when the construct was co-transfected with cloned pre-miR-125b or pre-miR-150 (**Figure 5A**). Although endogenous p53 level was decreased by over expressing miR-125b and miR-150, there was no change in p53 level either in the presence of exogenous miR-19a or miR-146a (negative control), which bears no predicted recognition site in the 3′UTR of p53 (**Figure 5B**). Taken together, these results show that p53 is specifically targeted by miR-125b and miR-150. Report that expressions of miR-125b and miR-150 are decreased in ST*Hdh^Q111^*/^Q111^ cells [**33**] and p53 is one of the targets of these two miRNAs provides an explanation for the increased expression of p53 in these cells. If this down regulation of miR-125b and miR-150 are confirmed along with increased p53 in the post mortem brains of HD, then it may explain the cause for elevated p53 and its role in HD pathogenesis as observed in other studies [4], [10], [11].

NFkB is known to resist apoptosis induction [54], [55] while p53 in general, is a well-known inducer of apoptosis. Thus down regulation of RelA/NFkB and increased expression of p53 as observed in ST*Hdh^Q111^/Hdh^Q111^* cells, a cell model of HD, if replicated in HD patients could be one of the mechanisms of enhanced apoptosis observed in HD as reviewed by Imarisio et al., 2008 [1]. The differential pattern of expression of the two transcription factors NFkB and p53 in ST*Hdh^Q111^/Hdh^Q111^* cells, prompted us to search for any relationship that might exist between the expressions of these two transcription factors. We observed an inverse correlation between the levels of p53 and p65 sub-unit of NFkB (RelA/NFkB) in neuronal cells. Exogenous expression of p53 and stabilization of p53 by 5-FU treatment decreased the expression of RelA/NFkB and activity of NFkB. Reducing the expression of p53 either by siRNA or by expressing exogenous miR-150 increased NFkB activity as detected by the luciferase assay. It is unclear what exactly mediates such functional inverse relationship. CREB binding protein (CBP) is known to interact with both p53 and RelA/NFkB and depending on the preference either induces or prevents apoptosis. Sequestration of CBP to p53 may decrease NFkB activity [38]. Activated p53 is also known to induce NFkB DNA binding but at the same time suppresses its transcriptional activation [37]. This may provide an explanation for the decreased NFkB activity as observed in our studies. However, there is a report, which suggests for an activation of the transcription factor NFkB in response to apoptosis induced by p53 [41]. Besides, RelA/NFkB is also known to regulate p53 expression in tumor cells in response to hypoxia [56]. All these results show that p53 directly or indirectly regulates RelA/NFkB expression and activity of NFkB and thus the expression of miR-146a. The other possibility of direct interference of p53 on miR-146a expression could not be ruled out and requires further studies. Even though it is conceivable that p53 can modulate the activity of NFkB, how the expression of RelA/NFkB is compromised remains unknown.

**Figure 12:**
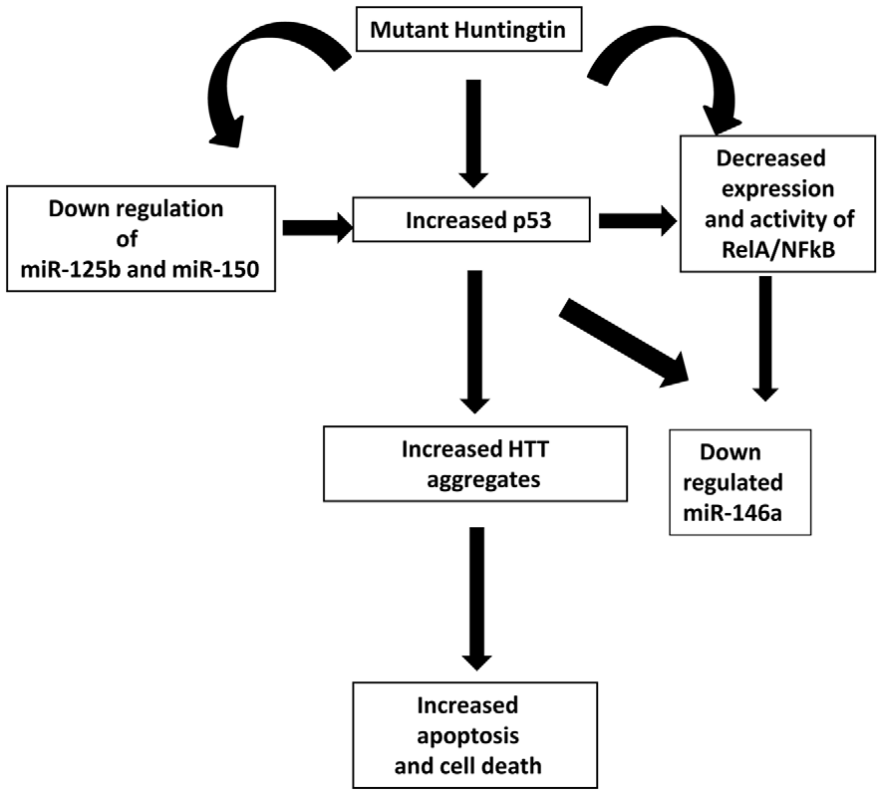
Proposed model for the involvement of RelA/NFkB, p53 and microRNAs in the regulation of cell death in HD pathogenesis. The model shows that mutant HTT modulates the expression of both p53 and p65 subunit of NFkB (RelA/NFkB) expression and activity and miR-146a, miR-125b and miR-150 expressions. Since miR-125b and miR-150 target p53, we postulate that in the presence of mutant HTT aggregates there is an initial decrease in miR-125b and miR-150 expression. These down regulated miRNAs lead to increased p53 level as observed in presence of aggregates. The elevated p53 then in turn, further increases mutant HTT aggregates and decreases NFkB/p65 expression (RelA/NFkB), NFkB activity and miR-146a expression.

Chaperones are known to protect mutant HTT aggregates possibly by preventing formation of aggregates. We have earlier shown that HYPK possesses chaperone like activity and reduces the aggregates formed by mutated N-terminal HTT coded by exon1 of the gene [45]. Exogenous expression of N-terminal HTT with 83Q increased the mutant HTT aggregates as have been observed by many authors including us [44]. In such condition, p53 expression was increased and RelA/NFkB expression was decreased (**Figure 9A**) similar to that obtained in endogenous ST*Hdh^Q111^/Hdh^Q111^* cells when compared with endogenous ST*Hdh^Q7^/Hdh^Q7^* cells. NFkB activity and expression of miR-146a, miR-125b and miR-150 were also reduced in such condition (**Figures 9B and 9C**). However, co-expression of HYPK together with mutant HTT exon1 reduced the aggregates, reduced p53 expression and recovered the activity of NFkB and miR-146a, miR-125b and miR-150 expressions (**Figures 10A–10D**). Similar results were also observed with Hsp70. Results obtained with ST*Hdh^Q111^/Hdh^Q111^* cells, a cell model of HD has been schematically represented in **Figure 12** to propose for a probable model showing the involvement of RelA/NFkB, p53 and miRNAs in the regulation of cell death in HD pathogenesis. The model shows that mutant HTT modulates the expression of p53 and p65 subunit of NFkB (RelA/NFkB), NFkB activity and miR-146a, miR-125b and miR-150 expressions. Since miR-125b and miR-150 target p53, we postulate that in the presence of mutant HTT aggregates there is an initial decrease in miR-125b and miR-150 expression. These down regulated miRNAs lead to increased p53 level as observed in presence of aggregates. The elevated p53 then in turn, further increases mutant HTT aggregates and decreases RelA/NFkB expression, NFkB activity and miR-146a expression.

In summary, we may conclude that in ST*Hdh^Q111^/Hdh^Q111^* cells, down regulation of miR-146a is mediated through RelA/NFkB. Increased p53 level in HD models could be mediated through down regulation of miR-125b and miR-150. p53 directly or indirectly regulates the expression of miR-146a. Identification of interplay between transcription factors and miRNAs regulating their targets remains one of the challenges in miRNA biology. Our investigation using HD cell lines provides important observations that miR-146a is regulated by p53 and RelA/NFkB and increased p53 could be mediated through down regulation of miR-125b and miR-150. It requires further studies to establish if such regulation plays any role in HD pathogenesis.

## Materials and Methods

### R6/2 mice

Ovarian transplanted hemizygote females carrying HD exon 1 gene with about 150 CAG repeats (strain name: B6CBA-Tg(Hdexon1)62Gpb/3J) were purchased from Jackson Laboratory and crossed with B6CBAF1/J males. The transgenic strain was maintained by crossing carrier males with CBA females. The genotyping was carried out using PCR as described previously by Mangirani et al., 1996 [47]. All animal experiments were performed according to the protocol approved by the Institutional Animal Ethics Committee of National Brain Research Centre, Manesar. Animals had free access to pelleted diet and water *ad libitum*. All efforts were made to minimize animal suffering. The transgenic mice along with their age-matched controls were anesthetized and then perfused with PBS containing 4% PFA in PBS, brain samples were collected and processed for paraffin embedding followed by cryosectioning with 20 µm thickness.

### ST*Hdh^Q7^/Hdh^Q7^* and ST*Hdh^Q111^/Hdh^Q111^* cells

ST*Hdh^Q7^/Hdh^Q7^* cells express full-length wild type HTT with 7Q (homozygous) while ST*Hdh^Q111^/Hdh^Q111^* cells express full length mutated HTT with 111Q (homozygous) from the chromosomal region and is considered as a model for HD. These cell lines were established from wild type and homozygous mutant Hdh knock in embryonic mice respectively [32]. Dr. Marcy E. MacDonald of Massachusetts General Hospital, USA, kindly provided these cells to us. ST*Hdh^Q111^/Hdh^Q111^* cells exhibit dominant HD phenotypes and indicate a disruption of striatal cell homeostasis by the mutant HTT protein, via a mechanism that is different from its normal activity (ST*Hdh^Q7^/Hdh^Q7^* cells). This cell model of HD has been extensively used for identifying molecular alterations in HD [57]–[64].

### Cell Culture

ST*Hdh^Q7^/Hdh^Q7^* and ST*Hdh^Q111^/Hdh^Q111^* cells, obtained from Dr. Marcy E. MacDonald, were cultured in DMEM (HiMedia, India) supplemented with 10% (v/v) heat inactivated FBS (Biowest, France), antibiotics penicillin/streptomycin PS 1% (v/v) and 400 µg/ml G418 (Invitrogen, USA) at 33°C in humidified condition and 5% CO_2_. HeLa cells were cultured in MEM (HiMedia, India), 10% (v/v) FBS (Biowest, France), 1% (v/v) PS at 37°C in humidified condition and 5% CO_2_. Growth conditions for HeLa cells [44] and growth conditions for Neuro2a cells [45] were similar to those which have already been published. Human derived neuroblastoma cells SH-SY5Y were cultured in DMEM (HiMedia, India), 10% (v/v) FBS (Biowest, France), 1% (v/v) PS at 37°C in humidified condition and 5% CO_2_.

### Antibodies and materials

Anti-p53 monoclonal antibody (1∶200, Clone DO7, Imgenex, USA) was used for immuno-precipitation. Anti-p53 polyclonal (1∶2000, Clone DO7, Imgenex, USA), anti-p65 monoclonal (1∶1000, MAB3026, Chemicon, USA), anti-GFP monoclonal (1∶4000, CATALOG No. 632375, Clontech, USA) and anti-β-actin monoclonal (1∶10,000, A2228, Sigma Chemicals, USA) antibodies were used for immunoblot analysis. Anti-HYPK polyclonal was custom made by providing purified HYPK protein [45] to Imgenex Biotech Pvt. Ltd, India (1∶1000, CP 40 07, Imgenex Biotech Pvt. Ltd, India). Anti-mouse IgG-HRP (1∶8,000) and anti-rabbit IgG-HRP (1∶6000) were purchased from GENEI, India and used as secondary antibodies. Aspirin was purchased from Central Drug House Laboratory Reagent, India and 5-Fluorouracil (5-FU) obtained from Sigma-Aldrich, USA was used.

### DNA constructs

Precursor miRNA-125b (Chr11: 121970465–121970552, - strand) and precursor miRNA-150 (Chr19: 50004042–50004125, - strand) were amplified by PCR from human genomic DNA and respectively cloned into pU61 Hygro (Genescript, USA) vector using BamHI and HindIII (NEB, USA) sites. The primers used were miR-125b-U6-F: 5′-CGCGGATCCGTCTCAAGAAAGAAAACATTG-3′ and miR-125b-U6-R: 5′-CCCAAGCTTAAAAACACCAAATTTCCAGGATGCAA-3′; miR_150_U6_F: 5′-CGCGGATCCCTCCCCATGGCCCTGTCT-3′ and miR_150_U6_R: 5′-CCCAAGCTTAAAAAGTCCCCAGGTCCCTGTCC-3′. Full-length human *p53* cDNA was obtained by PCR from human brain cDNA library and cloned into CFP vector using BamH1 and Sal1 sites. The primers used for cloning were p53_CFP_F: 5′-ACGCGTCGACGTGGAGCCGCAGTCAGATCCTA-3′ and p53_CFP_R: 5′-CGCGGATCCCAGTCTGAGTCAGGCCCTTC-3′. Full-length p65 subunit of NFkB (RelA), cloned into pLG3 vector was obtained as a kind gift from Dr. Susanta Roychoudhury, IICB, Kolkata. For knocking down p53, pSuppressorNeo p53 plasmid DNA containing p53 siRNA [65] construct (Imgenex, USA) was used.

For luciferase reporter assays, we cloned two fragments of the 3′ UTR of human p53 into pmiR-Report vector (Ambion, USA), one comprising of 150 bp (position 733–739) containing miR-125b recognition site and the other comprising of 136 bp (position 234–256) containing miR-150 recognition site. These regions were amplified by PCR from human genomic DNA and cloned in vector using the MluI and HindIII (NEB, USA) sites. The constructs were named p53-UTR1 and p53-UTR2 respectively. For NFkB1 (p50), 213 bp (position 145–359) of the 3′ UTR of NFkB1 containing no predicted recognition site for either miR-125b or miR-150 was cloned into the vector using SpeI and MluI (NEB) sites and was named p50-UTR. The following primers were used to generate the UTR constructs: p53-UTR1-F: 5′-CGACGCGTAAGGAAATCTCACCCCATCC-3′ and p53-UTR1-R: 5′-CCCAAGCTTAAGCGAGACCCAGTCTCAAA-3′; p53-UTR2-F: 5′-CGACGCGTGAGGAGGATGGGGAGTAGGA-3′ and p53-UTR2-R: 5′-CCCAAGCTTAAGTGGGCCCCTACCTAGAA-3′; p50-UTR-F: 5′-GGACTAGTTTGGCTTCCTTTCTTGGTTC-3′ and p50-UTR-R: 5′-CGACGCGTGGCGACCGTGATACCTTTAAT-3′. For functional assay of NFkB promoter, the plasmids NFkB luciferase, containing multiple copies of NFkB response elements (NFkB-RE) and Gastrin luciferase containing the promoter sequence of Gastrin cloned in pGL3 vector were obtained as a kind gift from Prof. Susanta Roychoudhury, IICB, Kolkata.

In order to induce poly Q mediated aggregation in ST*Hdh^Q7^/Hdh^Q7^* cells, Neuro2a cells, SH-SY5Y cells and HeLa cells, N-terminal HTT with 83Q coded by exon1 of the gene cloned into Ds Red-C1 vector was used. For removal of such aggregates with the help of chaperones in ST*Hdh^Q7^/Hdh^Q7^* cells, DNA constructs containing full-length Hsp70 cloned into EGFP-C1 vector and N terminal of HYPK also cloned into EGFP-C1 vectors were used. For control experiments, empty vectors DsRedC1 and pEGFPC1 (both from BD Biosciences, USA) were used.

### RNA preparation

Total RNA was prepared from cultured cells using TriZol Reagent (Invitrogen, USA) according to manufacturer's protocol. RNA samples were quantitated using Biophotometer (Eppendorf, Germany).

### RNA extraction from paraffin embedded tissue samples

RNA was isolated from tissue sections routinely processed by fixation and paraffin embedding that does not completely degrade RNAs. Further, it is suggested that RNA fragments of around 100 bases in length or more are still present even in organs fixed at later stages after removal and also in organs very rich in RNase such as the pancreas [66]. We isolated RNA from paraffin embedded tissue samples of R6/2 mice following the protocol described by Santa et al., 1998, Korbler et al., 2003 [67], [68]. Using this method many investigators studied expressions of coding sequences of DNA like β-actin, Ikaros and Aliolos from paraffinised lymph node sections of patients with malignant disorders of the lymphopoietic system (Hodgkin's and non-Hodgkin's lymphoma) [68]. There have been previous reports of studies on miRNA expression patterns from total RNA isolated from formalin-fixed paraffin-embedded (FFPE) tissues and frozen cells [69], [70], [71].

In brief, isolation method for RNA from paraffin embedded tissues consists of the following steps: De-paraffinization: For RNA extraction from tissue sections obtained from R62 mice, two sections each of 20 µm thickness were taken per 1.5 ml eppendorf tube. The sections were deparaffinised by two rinses in xylene for 5 min each at room temperature followed by two centrifugations at room temperature for 10 min each at 10,000 g. Rehydration: After paraffin solubilization, a rehydration step was introduced where the supernatants from the previous step were carefully removed and the pellets were successively washed with 1 ml of absolute ethanol and 1 ml of 95% ethanol in DEPC water. After each step the tissue was collected by centrifugation at 10,000 g for 10 min. Protein digestion: After the final wash, alcohol was aspirated and the tissue pellets were air dried in a thermoblock at 37°C and re-suspended in 500 µl of digestion buffer (10 mM NaCl, 500 mM Tris, pH 7.6, 20 mM EDTA and 1% SDS). To obtain purified RNA, tissue proteins were removed by adding 500 µg/ml of the proteolytic enzyme proteinase K. The sections were then incubated at 45°C for 16 hours (overnight). Prior to RNA purification, proteinase K was inactivated at 100°C for 7 min in order to nullify its effects on PCR. RNA extraction: Total RNA was then extracted from these tissue sections by using Trizol reagent and following manufacturer's protocol. Concentrations of total RNA was measured and total RNA was used to measure expression levels of genes like β-actin (contol), p53 and RelA/NFkB (p65 sub-unit of NFkB) and microRNAs like miR-150, miR-125b, miR-146a and miR-17-5p after making the cDNA.

### Quantitative real time for miRNAs and their analysis

For Real time quantitation of microRNA, 100 ng of total RNA was taken for cDNA preparation using mirna specific stem-loop primers (ABI), Mulv-Reverse transcriptase (Fermentas), RNase inhibitor (Fermentas) and dNTPs. cDNA was then subjected to the procedure as described in [33]. We earlier confirmed miR-17-5p expression to remain unaltered in various conditions and cells including HD cell model viz., ST*Hdh^Q7^/Hdh^Q7^* and ST*Hdh^Q111^/Hdh^Q111^* cells and thus it was used as an endogenous control in our laboratory [33]. Besides, miR-17-5p has also been found to be useful as a suitable endogenous control in other studies as well [72]. In the present work also the expression of miR-17-5p was found to remain unchanged in various conditions and cells used. Thus, miR-17-5p was used as endogenous control to calculate fold change in all RT-PCR studies. The fold changes were calculated in accordance with SDS software V 2.0.

### Real time PCR for mRNAs analysis

For real time of mRNAs 1 µg of total RNA was subjected to DNase treatment (Sigma) followed by cDNA preparation using random hexamer primer (Fermentas), dNTPs and MuLv- Reverse transcriptase (Fermentas) following the procedure described in [33]. The primers used for RT-PCR were: p53_expression_F: 5′-TCCCCCTTGCCGTCCCAA-3′, p53_expression_R: 5′-CGT- GCAAGTCACAGACTT-3′; RelA(mse)_expression_F: 5′-GGCCTCATCCACATG- AACTT-3′, RelA(mse)_expression_R: 5′-CACTGTCACCTGGAAGCAGA-3′; and Actin-β_F: 5′-TCCTGTGGCATCCACGA- AACT-3′, Actin-β_R: GAAGCATTTGCGGTGGAC. Yield of the PCR products was estimated from the integrated optical density (IOD) using Image Master VDS software (Amersham Bioscience, UK). Data show mRNA expression levels relative to those of *β-actin*; the former was then normalized to control expression levels for each experiment.

### Luciferase assays

For reporter luciferase assay, ST*Hdh^Q7^/Hdh^Q7^* and ST*Hdh^Q111^/Hdh^Q111^* cells were plated the day before transfection at 5×10^4^ cells per well in 24-well plates (Nunc, USA). The following day, 50 ng of p53-3′UTR in pmiR-Report luciferase vector was transfected into cells using Lipofectamine 2000 (Invitrogen) according to the manufacturer's instructions. 24 hours later, luciferase assays were performed (Sirius Luminometer, Berthold detection systems, USA) using Luciferase Reporter assay system (Promega) according to the manufacturer's protocol. 3 µg of total protein was taken for each assay. Transfection efficiency was normalized by co-transfecting cells with GFP-C1 and measuring the Fluoresence at 510 nm (Fluoromax-3, Jobin Yvon Horiba, USA). The luciferase activity of cloned constructs in ST*Hdh^Q111^/Hdh^Q111^* and ST*Hdh^Q7^/Hdh^Q7^* cells were normalized to the activity of empty vector (pmiR-Report luciferase vector), to nullify difference in protein synthesis levels between the wild type ST*Hdh^Q7^/Hdh^Q7^* and ST*Hdh^Q111^/Hdh^Q111^* cells. The experiments were carried out in triplicate. For over expression studies, 200 ng of pmiR-Report with desired clone and 300 ng of cloned pre-miR-125b or pre- miR-150 were co-transfected and luciferase assay was done following the same procedure.

For functional assay of NFkB response element and Gastrin promoter construct, 1×10^5^ ST*Hdh^Q7^/Hdh^Q7^* cells and 1.5×10^5^ ST*Hdh^Q7^/Hdh^Q7^* and ST*Hdh^Q111^/Hdh^Q111^* cells were plated a day before transfection per well in 6-well plates. The following day, 1 µg of NFkB response element construct and Gastrin luciferase construct were separately transfected. 24 h or 48 h post transfection, cells were collected and luciferase assays were performed as discussed above. For inhibiting NFkB activity, cells were treated with 2 mM aspirin 24 hours prior to transfection. For over expression studies 1 µg of p53-CFP and 1 µg of empty vector (CFP) were separately co-transfected with NFkB response element construct in ST*Hdh^Q7^/Hdh^Q7^* cells and for knocking down p53, 1 µg of p53 siRNA construct was transfected in HeLa cells (data not shown) and ST*Hdh^Q7^/Hdh^Q7^* cells and 1 µg of miR-150 was transfected in ST*Hdh^Q111^/Hdh^Q111^* cells along with 1 µg of NFkB luciferase construct. Luciferase assay was performed in each case following the same procedure as discussed above.

### Immunoblot analysis

Cells were washed with cold phosphate-buffered saline (PBS), scraped, pelleted by centrifugation and lysed on ice for 30 mins using RIPA lysis buffer (50 mM Tris-HCl pH 8, 1% NP40, 150 mM NaCl, 12 mM deoxycholic acid sodium salt, 0.1% SDS) with protease inhibitor cocktail (Thermo Scientific, USA). The supernatant collected after centrifugation (at 4°C for 15 min at 18,000 g) was estimated by Bradford assay (BioRad, Hercules, CA) according to manufacturer's protocol. The OD readings of the samples were measured at 595 nm in Biophotometer (Eppendorf). For immunoblots 30 or 60 µg of total protein, boiled in SDS PAGE sample buffer were run on 10% SDS-PAGE, transferred to PVDF membranes (Thermo Scientific, USA) and detected by immunoblottting with the indicated antibodies. Integrated optical density (IOD) of each band compared to individual loading control was measured using Image Master VDS software (Amersham Biosciences, UK).

### Co-immunoprecipitation assay

For co-immunoprecipitation assay, cells washed in cold PBS were lysed in co-immunoprecipitation (co-IP) buffer (50 mM Tris, pH 8.0, 150 mM NaCl, 1% NP40 and complete protease inhibitor cocktail) for 1 hour at 4°C with gentle mixing on an eppendorf rotor. Cell lysates were then centrifuged at 16,000 g for 15 min at 4°C and 50 µg of supernatant (total soluble extract) was used as an input for immunoprecipitation. For each experiment, 200 µg of the supernatant in 200 µl co-IP buffer was pre-cleared with agarose-protein G beads (GENEI, Bangalore, India) at 4°C for 2 hours with gentle shaking and then centrifuged at 1000 g for 5 min at 4°C. The beads obtained were washed twice with co-IP buffer, boiled in SDS PAGE loading buffer and used as negative antibody. The supernatant obtained was incubated overnight with 1 µg of p53 antibody at 4°C with gentle shaking. Next day, agarose-protein G bead was added to it and kept shaking for 6 hours at 4°C. The beads were precipitated by centrifuging at 18,000 g for 15 min at 48C, washed thrice by co-immunoprecipitation buffer, boiled with SDS–PAGE loading buffer and run on 10% SDS–PAGE and analyzed by immunoblotting technique with anti-p65 antibody.

### Statistical analysis

Statistical analysis was done with the help of Graphpad Software, QuickCalcs, (http://www.graphpad.com/quickcalcs/index.cfm). Student's t-test was performed between control and experimental values to determine their statistical significance.

## Supporting Information

Figure S1 **(A) hsa-miR-150 binds to the 3′UTR of human p53.** The position (234–256) in human p53-3′UTR predicted by miRBase as the recognition site for hsa-miR-150. Texts in blue indicate the ‘seed’ region. **(B). mmu-miR-125b binds to the 3′UTR of mouse Trp53.** (I) The position (413–435) in mouse p53-3′UTR predicted by RNAhybrid as the recognition site for mmu-miR-125b. Texts in blue indicate the ‘seed’ region. Their predicted stable RNA-RNA duplex formed by the binding of miR-125b to the 3′UTR of mouse Trp53 is shown in panel (II). The RNA strand in green represents mmu-miR-125b and the RNA strand in brown represents 413–435 of the 3′UTR in the mouse Trp53 transcript. **(C). mmu-miR-150 binds to the 3′UTR of mouse Trp53.** (I) The position (260–287) in mouse p53-3′UTR predicted by RNAhybrid as the recognition site for mmu-miR-150. Texts in blue indicate the ‘seed’ region. Their predicted stable RNA-RNA duplex formed by the binding of miR-150 to the 3′UTR of mouse Trp53 is shown in panel (II). The RNA strand in green represents mmu-miR-150 and the RNA strand in brown represents 260–287 of the 3′UTR in the mouse Trp53 transcript.
(TIF)

Table S1 **MicroRNA expression changes in 83Q DsRed transfected cells compared to controls.** Expressions of several miRNAs were studied in striatal regions of the brains of R6/2 mice and in four different cell lines exogenously expressing N-terminal HTT with 83Q coded by the exon1 of HTT gene and the results thus obtained have been indicated in the table. miR-17-5p was taken as endogenous control and fold change greater than 1.5 was considered as deregulated. The results obtained were compared with those found in HD cell model [33]. Texts in bold show names of miRNAs which maintained similar trend in their individual expression pattern in at least four of the six different models used for comparison. miR-125b and miR-150 were down regulated by more than 1.5 fold in five of the models including R6/2 mice whereas miR-146a was down regulated in all the models. Other miRNAs which showed a consistent expression pattern across the models were miR-100, miR-214, miR-299, miR-335, miR-34a and miR-148a. However, miR-148a which was up regulated in HD cell model [33] had been shown to be down regulated in all other models. The remaining miRNAs which were deregulated in HD cell model [33] have however showed heterogeneity in their expression pattern across the various models. The results obtained indicate that despite differences in miRNA expressions in various models, miR-146a, miR-125b and miR-150 were preferentially down regulated than others in the presence of poly Q aggregates.
(TIF)
