# Supplementary figures and images for: Regulation of miR-146a by RelA/NFkB and p53 in STHdhQ111/HdhQ111 Cells, a Cell Model of Huntington's Disease
Source: PLoS One. 2011 Aug 26;6(8):e23837. doi: 10.1371/journal.pone.0023837 (PMC3162608; doi:10.1371/journal.pone.0023837)

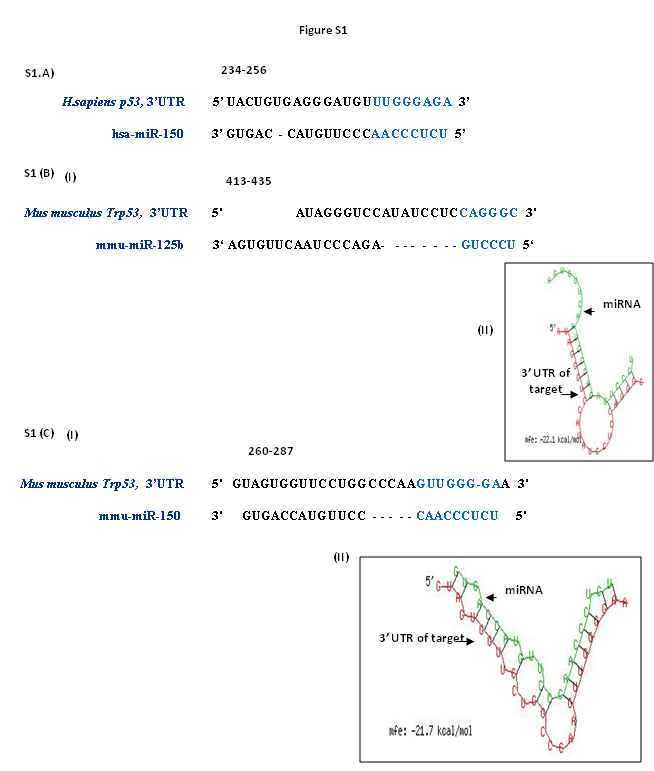

Supplement: Figure S1 — (A) hsa-miR-150 binds to the 3′UTR of human p53. The position (234–256) in human p53-3′UTR predicted by miRBase as the recognition site for hsa-miR-150. Texts in blue indicate the ‘seed’ region. (B). mmu-miR-125b binds to the 3′UTR of mouse Trp53. (I) The position (413–435) in mouse p53-3′UTR predicted by RNAhybrid as the recognition site for mmu-miR-125b. Texts in blue indicate the ‘seed’ region. Their predicted stable RNA-RNA duplex formed by the binding of miR-125b to the 3′UTR of mouse Trp53 is shown in panel (II). The RNA strand in green represents mmu-miR-125b and the RNA strand in brown represents 413–435 of the 3′UTR in the mouse Trp53 transcript. (C). mmu-miR-150 binds to the 3′UTR of mouse Trp53. (I) The position (260–287) in mouse p53-3′UTR predicted by RNAhybrid as the recognition site for mmu-miR-150. Texts in blue indicate the ‘seed’ region. Their predicted stable RNA-RNA duplex formed by the binding of miR-150 to the 3′UTR of mouse Trp53 is shown in panel (II). The RNA strand in green represents mmu-miR-150 and the RNA strand in brown represents 260–287 of the 3′UTR in the mouse Trp53 transcript. (TIF) [file pone.0023837.s001.tif]

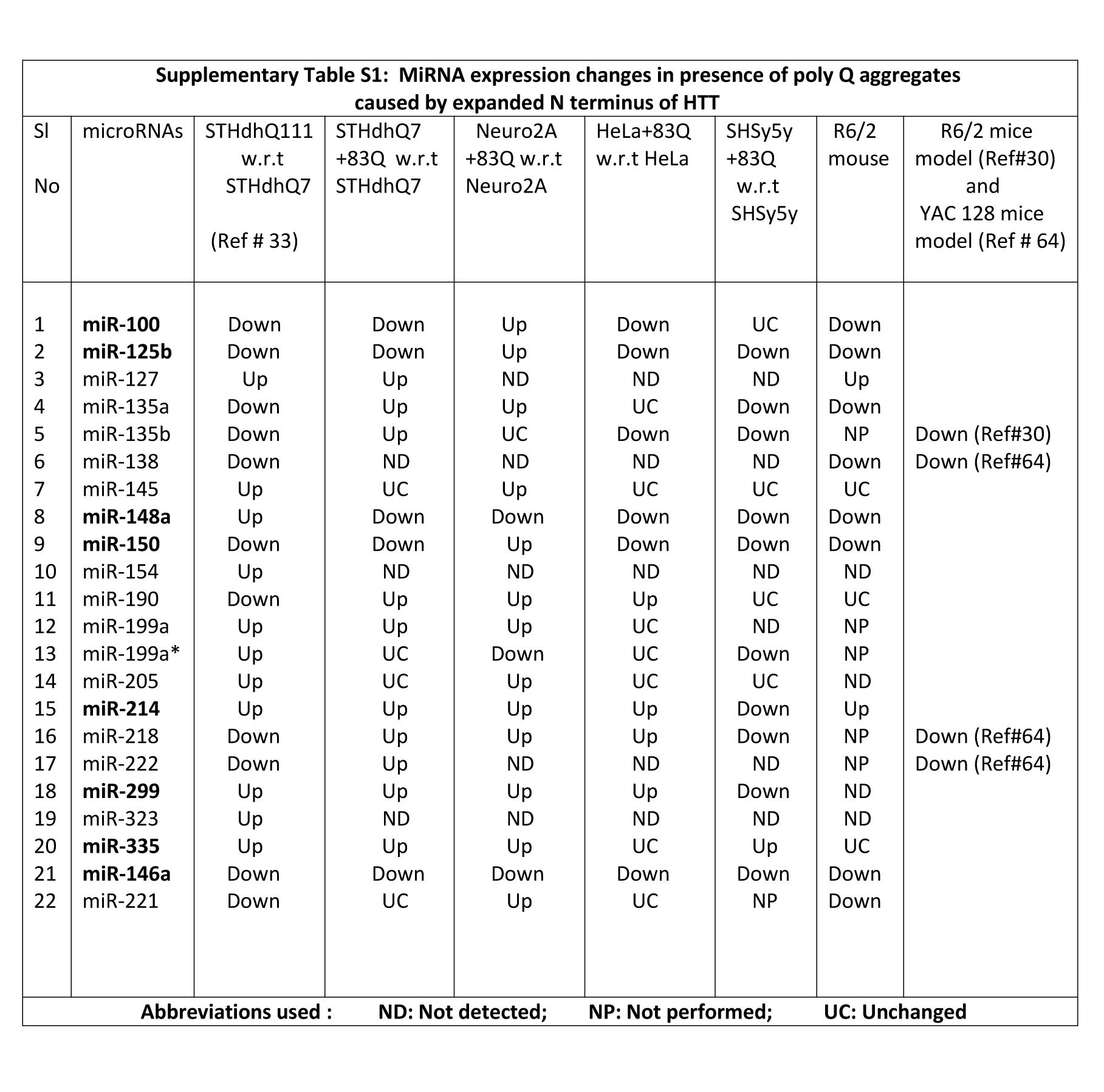

Supplement: Table S1 — MicroRNA expression changes in 83Q DsRed transfected cells compared to controls. Expressions of several miRNAs were studied in striatal regions of the brains of R6/2 mice and in four different cell lines exogenously expressing N-terminal HTT with 83Q coded by the exon1 of HTT gene and the results thus obtained have been indicated in the table. miR-17-5p was taken as endogenous control and fold change greater than 1.5 was considered as deregulated. The results obtained were compared with those found in HD cell model [33]. Texts in bold show names of miRNAs which maintained similar trend in their individual expression pattern in at least four of the six different models used for comparison. miR-125b and miR-150 were down regulated by more than 1.5 fold in five of the models including R6/2 mice whereas miR-146a was down regulated in all the models. Other miRNAs which showed a consistent expression pattern across the models were miR-100, miR-214, miR-299, miR-335, miR-34a and miR-148a. However, miR-148a which was up regulated in HD cell model [33] had been shown to be down regulated in all other models. The remaining miRNAs which were deregulated in HD cell model [33] have however showed heterogeneity in their expression pattern across the various models. The results obtained indicate that despite differences in miRNA expressions in various models, miR-146a, miR-125b and miR-150 were preferentially down regulated than others in the presence of poly Q aggregates. (TIF) [file pone.0023837.s002.tif]
